# Centre assessment grades in 2020: a natural experiment for investigating bias in teacher judgements

**DOI:** 10.1007/s42001-023-00206-x

**Published:** 2023-05-15

**Authors:** Louis Magowan

**Affiliations:** Department of Methodology, London School of Economics and Political Science (LSE), London, UK

**Keywords:** Quantitative education research, Bias in teacher judgements, Educational inequality during COVID-19, Machine learning for causal inference, GRADE data, Intersectionality

## Abstract

The COVID-19 pandemic meant that, in 2020, students in England were unable to sit their examinations and instead received predicted grades, or “centre assessment grades” (CAGs), from their teachers to allow them to progress. Using the Grading and Admissions Data for England (GRADE) dataset for students from 2018 to 2020, this study treats the use of CAGs as a natural experiment for causally understanding how teacher judgements of academic ability may be biased according to the demographic and socio-economic characteristics of their students. A variety of machine learning models were trained on the 2018–19 data and then used to generate predictions for what the 2020 students were likely to have received had their examinations taken place as usual. The differences between these predictions and the CAGs that students received were calculated and then averaged across students’ different characteristics, revealing what the treatment effects of the use of CAGs were likely to have been for different types of students. No evidence of absolute negative bias against students of any demographic or socio-economic characteristic was found, with all groups of students having received higher CAGs than the grades they were likely to have received had they sat their examinations. Some evidence for relative bias was found, with consistent, but insubstantial differences being observed in the treatment effects of certain groups. However, when higher-order interactions of student characteristics were considered, these differences became more substantial. Intersectional perspectives which emphasise interactions and sub-group differences should be used more widely within quantitative educational equalities research.

## Introduction

This study will look at education in England in the 2020 academic year, and how it was disrupted by the COVID-19 pandemic, as a lens through which to examine how educational inequalities may result from the use of teacher judgements in the assessment of academic ability. For context, on the 20th of March 2020, the Secretary of State for Education decided to close all schools and colleges in England to try and slow the spread of COVID-19 [11]. Furthermore, it was announced that summer examinations for that year would be cancelled and that General Certificate of Secondary Education (GCSE), Advanced Subsidiary Level and Advanced Level (AS and A-Level) students (these are all UK secondary school-leaver examinations) would instead receive calculated grades to allow them to progress into the labour market and higher education [21]. Following this decision, teachers were instructed to produce centre assessment grades (CAGs) for their students to represent what they think the students would have achieved had schools remained open and exams gone ahead [35].

It is important that this process was as fair as possible, as substantial educational inequalities already exist in the UK. In terms of free school meals (FSM), which are a proxy measure for socio-economic status (SES), the results for the 2019 GCSEs showed that only 22.5% of students who were eligible for FSM received grade 5 or above in English and Maths, compared with 46.6% of students who were not eligible [10]. In other words, lower SES students tend to perform worse academically. Similarly, clear ethnic divisions can be seen in the results with 37.8% of Black, 42.4% of White and 76.3% of Chinese students achieving those grades. Educational inequalities can also be found in terms of gender, whether English is an additional language (EAL) for a student, and whether the student has special educational needs (SEN) [10, 12, 24]. In the interests of brevity, these characteristics (SES, ethnicity, gender, EAL and SEN) will be referred to as “protected characteristics”^1^. This study will focus primarily on SES and ethnicity, as there is strong evidence that they are some of the most important contributing factors to educational inequality. For example, Strand [44] finds the impact of ethnicity and SES to be three and nine times larger, respectively, than the impact of gender on mean attainment of 14-year-olds in the UK.

There are both intrinsic and extrinsic reasons why such inequalities should be reduced [3]. Intrinsically, one might deem an extremely large gap between those with the highest educational attainment and those with the lowest to be undesirable – particularly if that gap is delineated along the lines of a characteristic such as ethnicity or SES. Extrinsically, there are many consequences of educational inequality that can make its reduction worthwhile. Educational inequalities in younger years can propagate with age and certain poor-performing students may not have access to the same range of subjects (e.g., Higher tier GCSEs in the UK) as their better-performing counterparts. Poor-performing students may also find they are unable to progress as far as they would like with their education, such as into university/higher education, or, in the UK, to their A-Levels. This can have material effects on their social mobility, labour market participation and even lifetime earnings [50]. Indeed, it has been shown that, across a range of countries, making education distributions more equal plays a significant role in making income distributions more equal [19]. Educational equality begets income equality and other external benefits.

Given the substantial inequalities already alluded to, the study of the CAG process could be regarded as worthwhile in its own right – as it is important the process was as equitable as possible. However, drawing on a case study typology [47], the CAG process can be thought of as a subject that helps to explicate the object of the use of teacher judgements in the assessment of academic ability. Appreciating the CAG process as a case in this way gives the study relevance beyond the summer 2020 exams that were cancelled in England. Furthermore, given how frequently teacher judgements are used to assess academic ability, it is important to understand how they may or may not be biased according to student’s protected characteristics. For example, in the UK teacher judgements are used as the basis of the predicted grades that A-Level students rely on in their applications to universities [49]. They also inform various Key Stage assessments, including being a component in the Key Stage 2 assessments that determine a pupil’s transition from primary to secondary school [43]. Teacher judgements also play a role in determining academic progression in many educational settings outside the UK [31, 48].

The CAG process has created a unique opportunity for investigating how teacher judgements may be biased. Indeed, the fact that they were awarded to all English students in 2020 has resulted in the largest dataset on teacher grading judgements that are available in the UK [45]. Moreover, it has created a natural experiment. Natural experiments, to use causal inference parlance, are observational studies in which some naturally occurring phenomena allows us to regard the assignment mechanism of some treatment to units as “as if” or virtually random [14]. In this instance, 2018, 2019 and 2020 English GCSE students can be regarded as essentially homogenous (see descriptive statistics section), except that the 2020 students received an exogenous treatment – examinations being cancelled and replaced with CAGs.

This study aims to exploit this natural experiment to assess causally how the use of teacher judgements in CAGs impacted students of various protected characteristics. A range of models will be trained and evaluated on 2018–19 data (and will be discussed in greater detail in the methodological section). The model with the highest predictive accuracy will then be used to generate predictions of GCSE examination grades for students in 2020. These predictions will then be compared with the CAGs that students of different protected characteristics received (e.g., a Chinese student; a low SES student; a low SES, Chinese student etc.), thereby throwing any causal impacts of the use of CAGs/teacher judgements into relief.

## Literature review

### Psychology of bias: stereotyping

Before considering the potential evidence for bias in teacher judgements, it is helpful to give a theoretical justification for it. In general terms, social bias can be classified into one of three forms [13]:

1. Prejudice: Individual-level attitudes which create or maintain hierarchical status relations between groups (can be subjectively positive or negative).
2. Discrimination: Behaviour that creates or reinforces an advantage for a group/group-member over another group/group-member.
3. Stereotyping: Beliefs about the characteristics and attributes of a group and its members that shape how people think about and respond to the group.

It is hoped, at least in a UK context where educational equality commissions and standards are well-established, that any bias that may arise in teacher judgements is primarily due to implicit, unconscious stereotyping rather than explicit discrimination or prejudice. That stereotyping is the *main* component of bias in teacher judgements would be hard to verify, however, Campbell [5] does find evidence that stereotypes according to income-level, gender, SEN, and ethnicity all play a part in forming biases in teacher judgements. Using data from the Millennium Cohort Study, Campbell demonstrates that certain categories of student were less likely to be judged “above average” by their teachers in terms of reading and maths ability when compared to students of other categories—despite having scored similarly in reading and maths tests. Even if stereotyping is not the main component of bias, it clearly plays a significant role.

There are several schools of thought on the psychological processes behind how stereotypes are formed and maintained. Some stereotypes stem from accurate, real group differences—accurate, at least, in terms of the local reality of the person who perceives them [23]. However, much psychological literature emphasises pathways in which stereotypes can be formed independently of any real, group differences. A widely cited example of such a pathway is that of the “self-fulfilling prophecy” [39]. This is the idea that the expectations teachers hold for their students can cause the students to alter their behaviour such that they end up aligning with their teachers’ expectations. Initially, there may have been no real, group differences in the academic performances of the students – but the teachers’ expectations manifest one, thereby maintaining the stereotype. Other, more recent literature on stereotypes highlights their interactive nature [26]. Stereotypes and other individuating factors (such as behaviour or personality) are not processed serially. Instead, each piece of information is combined by the mind in a simultaneous, rather than additive, fashion. In this way, stereotypes can jointly influence each other, interacting to produce a distinct impression about someone. Given that stereotypes are likely an important contributor to teacher bias and have themselves been shown to be influenced by protected characteristics, any study of teacher bias should therefore pay attention to interactions between protected characteristics.

### Examples of bias in teacher judgments

A considerable amount of research on bias in teacher judgements has been conducted both in the UK and internationally. In a sample of 53 Flemish primary schools, Boone and Van Houtte [4] found that, regardless of prior achievement, pupils of lower socio-economic backgrounds were less likely to be advised by their teachers to enroll in academically oriented school tracks than their counterparts from higher socio-economic backgrounds. Similar results have been found within the Dutch context. In a study of 500 classes [48] it was found that teachers held higher academic expectations for students from more affluent families, even after controlling for the students’ performance. Higher expectations were also observed for girls in this study. Some evidence of SES impacts on teacher judgements has also been found in the UK. Murphy and Wyness’ [33] study of A-Level predicted grades found small but significant differences in the predicted grades received by high-achieving students, depending on their school type and SES. Among high-achieving students, state school students received 0.16 fewer predicted grade points than their privately educated counterparts and low SES students got 0.059 fewer predicted grade points than their higher SES counterparts. Based on these studies, gender, school type, and particularly SES would seem to have an impact on teacher judgements—although the SES effect may be working interactively with prior attainment.

SES and gender impacts on teacher judgements are not found in all literature on the topic, however. Jussim and Eccles’ [25] study of 100 teachers in the US found no evidence of teachers being biased against students from lower social class backgrounds, or against either gender. They also found no evidence of bias against African American students. However, other US-based investigations would seem to contradict this last result. Zucker and Prieto [52] asked 280 special education teachers to indicate whether placement into special education classes would be appropriate for a given set of children. They found evidence of a significant main effect for ethnicity- with special class placement being deemed more appropriate for Mexican American children than for white children. Shiner and Modood’s [42] investigations of UK A-Level predicted grades contradicts both two previous studies yet again – instead of finding a negative or no ethnic bias in teacher judgements, they found evidence of a positive one. They found that while teachers’ A-Level predictions generally tended towards optimism when wrong, this was particularly the case for ethnic minorities. On average, predicted scores were 2-grade points higher than the final, achieved scores for White candidates, compared with 5, 4 and 3 points higher for Black Caribbeans/Black Africans, Indians/Pakistanis/Bangladeshis, and Chinese candidates, respectively, in their sample. Indeed, Murphy and Wyness’ [33] study, dealing with a similar sample of UK students’ A-Level predictions, reveals a similar pattern – with Asian and Black students being more likely than other ethnicities to be severely^2^ overpredicted.

Overall, the role that ethnicity plays in affecting teacher judgements seems to be unclear, though it may be a contributor to relative, positive bias for certain students in a UK context. It should also be noted that much of the existing UK research focusses solely on AS/A-Level students, as this was where teacher prediction data was most readily available previously. However, AS/A-Levels are not compulsory for all students like GCSEs are and so are not as a representative of the UK population. For example, there are SES differences between GCSE and AS/A-Level cohorts, with low SES students being significantly less likely to progress to AS/A-Level [41]. Studying GCSE teacher prediction data rather than AS/A-Level data could help ensure results are more generalisable to the UK population. Furthermore, given that educational inequalities can be seen even in early childhood and propagate with age [6], it could be worthwhile to consider students of a younger age range than AS/A-Level students – as GCSE students are.

### Meta-analyses of bias in teacher judgements: contradictory findings

Given the large amount of research on the topic of bias in teacher judgements and the contradictory findings reported in a lot of them, it can be helpful to instead consider meta-analyses of the topic. Dusek and Joseph’s [15] meta-analysis of 77 studies found that both social class and race were significant bases in how teachers formed expectancies about their students’ academic ability and that gender was not. Middle SES students were expected to perform better academically than low SES students and White students were expected to perform better than Black or Mexican students. Tenenbaum and Ruck’s [46] review of 32 US studies also found differences in terms of race for the expectations that teachers held for their students. They found small, but statistically significant effects that suggested teachers held lower expectations for African American and Latino/a children than for European American children.

While Dusek and Joseph’s results on the importance of ethnicity in teacher judgements would seem to be corroborated by this second meta-analysis, their results around the impact of gender are contradicted by a third. A review of 30 studies, mainly from the US and the UK, [20] found that there was strong evidence of bias in teacher judgements in terms of both gender and SEN. Indeed, within many of the studies included in the three previous meta-analyses (and in the works reviewed earlier in this study) many of the magnitudes and even signs of coefficients for various protected characteristics with teacher judgements seem to disagree. Even the conclusions *between* the reviews/meta-analyses themselves are not consistent, as was noted in Ofqual’s [28] recent literature review on the topic. Something that is consistent between these literature reviews and the studies they discuss, however, is that few, if any, of them have had access to a dataset of teacher judgements that is as large or as representative as that provided by the CAG process. The analysis of such a dataset and the natural experiment context it is set in could help bring greater clarity to an area of research that is full of contradictions. Furthermore, much of the existing literature only considers a small number of protected characteristics at a time. However, the dataset that is available around the CAG process is extremely rich and has many features of a protected characteristic in it. This means that potential inequalities in teacher assessments can be explored across a larger number of features at the same time.

### Prior research on centre assessment grades: an intersectional perspective

Some research into the use of CAGs has already been conducted. In an analysis by He and Black [22], exam results for the 2020 year were compared with those of the preceding year. GCSEs were on average three-fifths of a grade higher in 2020.^3^ This suggests that the CAG predictions were optimistic overall. This is to be expected as previous research on the UK university application system (which relies heavily on teacher-predicted grades) has shown as much as 75% of applicants in 2013–15 received lower grades than they were predicted [Wyness, 51]. An interesting difference between these studies, however, is that while He and Black found the correlations between prior attainment and grades to be the same in 2019 as in 2020 – Wyness’ findings somewhat contradict this. Her study showed that high-achieving disadvantaged students were more likely to be under-predicted than their more advantaged counterparts. Additionally, low-achieving students (who were disproportionately low SES) were far more over-predicted. This could imply that there is an interaction between SES and prior attainment that isn’t being considered in He and Black’s analysis.

Interactions such as this are why an “intersectional” perspective on the CAG process could be helpful. Intersectionality is a concept derived from feminist theory that views categories of ethnicity, class, gender, etc. as interrelated and mutually shaping one another [8]. Though the concept has not been frequently applied within quantitative educational research, it can be highly appropriate if the underlying data is rich and granular [7], as the CAG data is. An intersectional approach emphasises how different types of (dis)advantage are not the same for everyone who experiences them and stresses the importance of interactions and sub-group differences, rather than just the main effects of e.g., protected characteristics. Bias in teacher judgements may operate in complex ways, which may not be noticed if viewed in purely additive terms.

That teacher judgements used for CAGs were in fact biased, cannot be assumed, however. In fact, two key Ofqual (UK examinations watchdog) investigations of the topic concluded that systematic bias was unlikely. The first investigation, a student-level equalities analysis, did not find evidence of bias against students in terms of their protected characteristics [27]. The second study looked more directly at the use of teacher judgements, trying to determine if the factors related to grades in 2020 were different from those related to grades in previous years in any consistent way [45]. Overall, grading patterns between 2020 and previous years were found to be similar – with only minor differences in the relationships between student and centre-level features with grades. While Stratton, Zanini and Noden [45] do consider some interactions^4^ in their analysis, they are at most two-way interactions – and many possible two-way interactions of protected characteristics are left unexplored. Higher-order interactions are not considered in Lee, Stringer and Nadir’s [27] work either. By drawing on an intersectional perspective and considering more (and higher order) interactions, biases could potentially be revealed that are nuanced, complex and would otherwise be hidden. Furthermore, to the best of the author’s knowledge, no non-Ofqual studies on the topic have been conducted. Ultimately, given the size and richness of the dataset, and the significance of the subject matter, it is important that the CAG process be investigated with a variety of perspectives and methodological tools.

### Research questions

This study has two research questions it seeks to answer. It uses a specific question around the subject or case [47] of the 2020 CAGs to address the object and a more general research question on the use of teacher judgements in the assessment of academic ability. Importantly, these research questions do not assume anything about the presence or direction of bias in teacher judgements according to protected characteristics during 2020, leaving space for the detection of no bias.

**Object:** *Which, if any, and how do protected characteristics of students impact upon teachers’ judgements of their academic ability?*

**Subject:** *What were the total grade point differences for English students of different protected characteristics between the CAGs they received and the grades they were likely to have received in 2020 had COVID interruptions not occurred?*

## Methodology

### Data collection

This study uses secondary data from the Grading and Admissions for England (GRADE) data-sharing project that is available through the Office for National Statistics’ (ONS) Secure Research Service (SRS). This is joined, student-level data taken from Ofqual and the Department for Education’s (DfE) National Pupil Database (NPD) which contains anonymised examination results, demographic information, and prior attainment indicators for English students. The full GCSE datasets for the 2018–2020 years will be considered. The analysis could also have been extended to cover summer 2021, as teacher assessments were used to replace exams then too [37]. However, pupils received in-person teaching for even less of that year than in 2020. Analysis of those assessments would likely be impacted by the differences in home learning environments [18] of pupils (access to private tuition, computers, internet)—on which data is not readily available. Similarly, the analysis does not extend back further than 2018. There have been major GCSE reforms since then [34] which have meant changing curricula and marking schemes. In restricting the analysis to 2018–2020, the data should be reasonably comparable across years.

The highly sensitive nature of the GRADE data was a key constraint for this study. To ensure non-disclosure, results could only be shared in aggregated form, and only if they belonged to a sub-group of at least 100 students.

### Data pre-processing

Bearing in mind the limited time and computational resources for this study (Appendix D), and the need for interpretability, several variables needed to be filtered out or collapsed into fewer categories. Many of these steps are outlined in Table 2. There were also, however, some pre-processing steps involving variables that were not used for analysis/prediction that are outlined in Appendix F.

Only GCSEs that had been reformed since 2018 were considered, though this still covers many of the most popular subject choices [34]. Additionally, only results for students who took at least 8 GCSEs including English and Mathematics^5^ were included. The data was then split into a control group of 2018–2019 data and a treatment group of 2020 data.

Splitting the data in this way acts as the “as if” random assignment mechanism that forms the basis of natural experiments [14]. COVID happened in 2020 and GCSE students in that year received the treatment of being given CAGs rather than sitting their exams but COVID could easily have happened in 2018 or 2019. This pseudo-randomisation balances, at least in expectation, all observed and unobserved pre-treatment covariates between treatment and control groups. This is where the internal validity of the study lies, as, provided the groups are homogeneous, it creates a reasonably strong basis for inference about the effects of the treatment on the students within the dataset [38].

As Table 1 shows, even after these pre-processing steps, the amount of data was considerable – with over 1 million results from over 100,000 students each year. Yet despite the size of the remaining sample, it had some important limitations such as some systematic missingness outlined in (Appendix F). The pre-processing steps taken also limit the representativeness of the sample, with each filtering out of certain categories of students reducing the external validity of results to students of other years, other nations,^6^ or indeed students from the same years that were dropped from the sample. However, it is hoped that this sacrifice in external validity is compensated by having a more manageable sample and more interpretable results.

**Table 1:** Sample sizes by year

Group	Year	Number of students	Number of GCSEs
Control	2018	112, 541	1, 017, 470
Control	2019	120, 483	1, 087, 738
Treatment	2020	115, 910	1, 028, 023

### Data analysis

A series of models were trained and tested on the control data, with the target variable being the grades that a student received for a given subject. All the variables in Table 2, except for GCSE / CAG, were used as predictors. The prediction task was constructed as a regression problem, though it could also have been made a classification problem. In other words, the models were attempting to predict continuous values in the range {0 $\le$ *x* $\le$ 9}, rather than discrete grades {0, 1, …, 9}. The regression approach was decided upon because predictions were aggregated into a continuous space anyway later in the analysis (averaged across given combinations of protected characteristics).

**Table 2:** Description of variables

Variable name	Variable description	Operationalisation	Variable type
Centre type	Type of school that pupil attended	–	Categorical: Academy, Sixth Form College, Secondary Selective etc
EAL	English as an additional language: Whether English is a native language for pupil	Filtered to remove pupils of unclassified EAL	Binary: EAL or not EAL
Ethnicity	Major ethnic group of pupil	Filtered to remove pupils of unclassified ethnicity	Categorical: Asian, Black, Chinese, Mixed, White or Any Other Ethnic Group
FSM status	Free school meal entitlement	Proxy measure for SES	Binary: FSM or no FSM
GCSE / CAG	Grade scores received by pupils through exams (2018–19) or teacher assessment (2020). Ranges from 9 (highest) to U (ungraded/fail). Key target variable for predictions	Recoded U grades to 0	Discrete, numeric: 0—9
Gender	–	Filtered to remove pupils with missing gender information	Binary: Male or female
IDACI Score	Proportion of children aged 0–15 living in income-deprived families within the lower super output area of their postcode	Proxy measure for SES (higher IDACI ≈ lower SES). Normalised between 0 and 1. Used in continuous form for prediction, recoded into quantiles for analysis	For prediction: Continuous, numeric, 0—1 For analysis: Ordinal: Very low, low, medium, high, very high
Prior Attainment	Standardised measure of prior attainment at key stage 2	Normalised between 0 and 1. Used in continuous form for prediction, recoded into quantiles for analysis	For prediction: Continuous, numeric, 0—1 For analysis: Ordinal: Very low, low, medium, high, very high
SEN Status	Special educational needs of pupil	Filtered to remove pupils of unclassified SEN status. Collapsed into SEN or No SEN	Binary: SEN or no SEN
Subject	Subject of the GCSE	Filtered to remove double award science (since this is predicted on a different scale)	Categorical: Mathematics, English Language, Chemistry etc
Tier	Tier of GCSE – foundation (max grade 5) or higher (max grade 9)	–	Binary: Foundation or higher

The predictive approach used has important consequences for how the accuracy of the models in this study can be evaluated. By predicting into a continuous space, the models could not be evaluated in terms of the proportion of predictions for which they were correct. Rather, they had to be evaluated in terms of how close they were to the correct grade. Specifically, the models were evaluated in terms of root-mean-square-error (RMSE). RMSE was selected as an evaluation metric due to its interpretability, with errors being given in the same units as that of the target variable, i.e., GCSE grade points. An 80:20 split of training to test data was used to evaluate the models with—the results of which can be seen in Table 3. Additionally, as a sanity check, feature importance analysis of the final model (see Appendix B) using Shapley values [30] was conducted to ensure that predictors were shaping predictions in ways that align with prior educational research.

**Table 3:** Model evaluations

Model	Train RMSE	Test RMSE
LGBM	1.320	1.357
Neural Network 32–32	1.400	1.399
Neural Network 32–64	1.401	1.401
OLS Linear	1.432	1.432
SVR Linear	1.435	1.434
SVM RBF	1.519	1.527

The range of models used included an:

- Ordinary least squares (OLS) linear model
- Radial basis function (RBF) and linear support vector regression (SVR) models^7^
- Neural network with two hidden layers (32 neurons in each layer)
- Neural network with two hidden layers (32 and 64 neurons in the first and second layer)
- Optuna hyperparameter-optimised Light Gradient Boosting Machine (LGBM)

The specific implementations for these models can be found in Appendix D.

As Table 3 shows, the model with the highest predictive accuracy (lowest test and train RMSEs) was the LGBM model. Substantively, this means that for a given prediction on the unseen, test data this model had an average error of 1.357 grade points—which is an important limitation to bear in mind when considering how the model may have performed on unseen, 2020 data. However, as the LGBM model was the most accurate, it was therefore selected as the final model. This meant that it was used to generate predictions for what the GCSE examination results for students across different subjects were likely to have been in 2020, based on the predictor features present in the treatment data. Importantly, the predictions were generated on a subject level, to allow for inter-subject effects and interactions. However, for the analysis of results, the model’s predicted grades, and CAG grades were summed to a student level. These steps were taken to try and make results more interpretable and so that a fuller set of results could be shared.^8^

**Fig. 1:**
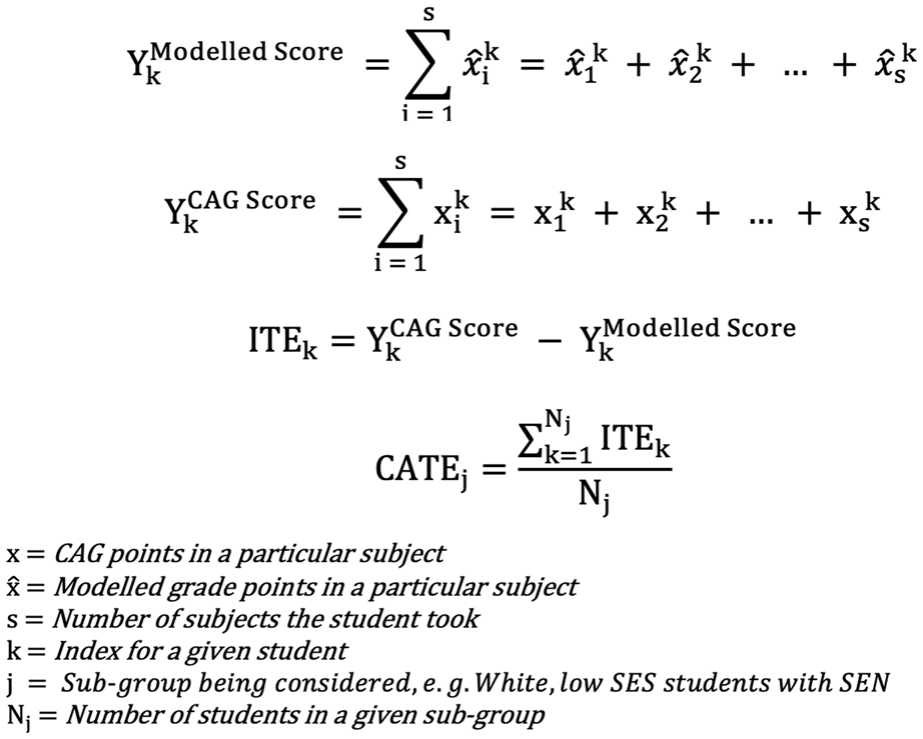
Formula | conditional average treatment effect for a given sub-group

The individual treatment effect (ITE) was estimated as the difference between a student’s total CAG score and their total modelled score. This approach relies on the potential outcomes framework [40] in which one tries to hypothesise what would have happened if an individual (student in each subject) had received both treatments (examinations and CAGs). The conditional average treatment effect (CATE) was then calculated as the mean of the ITEs of all students for a given sub-group (see Fig. 1) [1]. This calculation was performed over a range of grouping variables of varying orders. Additionally, the continuous IDACI and prior attainment variables were transformed into quantiles to make it easier to compare e.g., students of very low prior attainment with those of very high prior attainment.

Finally, due to the richness of the GRADE dataset, the vast number of sub-group combinations and the word-limit constraint of this project, it was necessary to restrict the number and detail of results shared. In this regard, an effort was made to either be guided by prior research on what key results should be, or by the content of the results themselves. In any case, the unabridged results are available to view in the dashboard linked in Appendix C. The dashboard also contains further analysis of intra-group ranges (differences between the largest and smallest CATEs within a group) across different levels of interaction.

## Results

### Descriptive statistics—control vs treatment

**Fig. 2:**
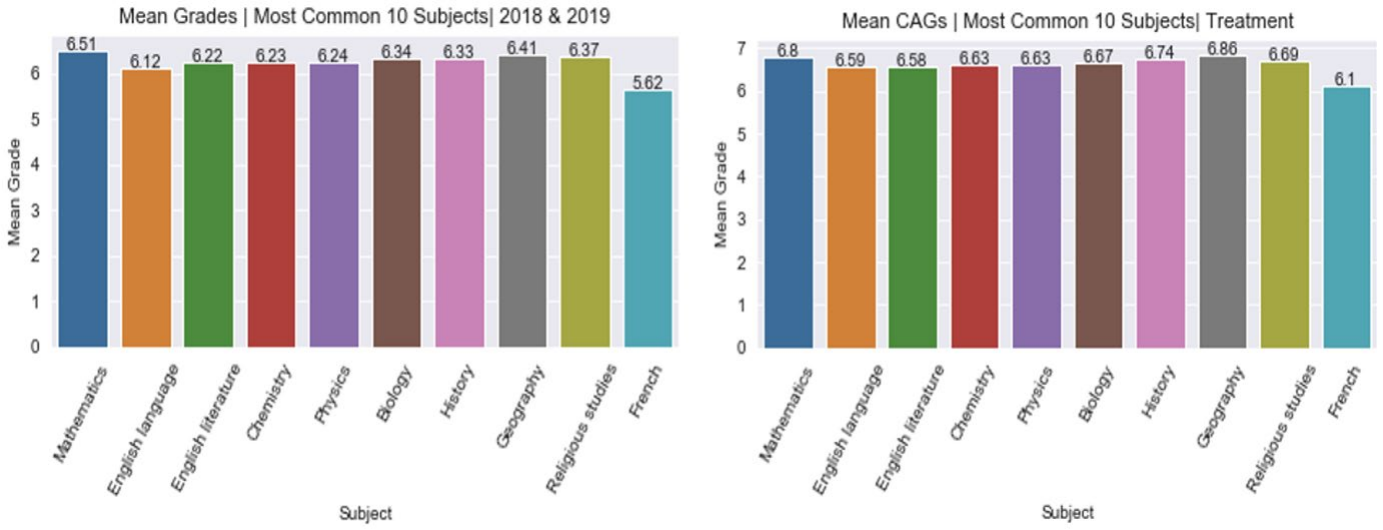
Mean grades by subject—control vs treatment

CAG grades were generally higher than the GCSE grades awarded in 2018–19, with the average CAG grade in this study’s sample being 6.622 versus only 6.210 for GCSEs in 2018–19. Indeed, each of the 10 most frequently taken subjects was graded more leniently, as can be seen in Fig. 2.

Tables 4 and 5 compare the mean values of continuous variables and proportions of categorical variables across the control and treatment groups. There are no major, substantive differences between the two. Appendix A looks at the proportions of students taking different subjects and finds no substantive differences across treatment or control either. Almost all the observed differences were statistically significant, however. This is to be expected given the extremely large sample size, as estimates become so precise that even small changes can be detected [29]. Overall, the control and treatment groups appear to be homogeneous in terms of their covariates.

**Table 4:** Continuous variables—checking for balance

Variable	Control mean	Treatment mean	*p* value	Conf. intervals of difference in mean
IDACI	0.152 (0.121)	0.155 (0.122)	< 0.001	[0.217%, 0.275%]
Prior Attainment KS2 Score	0.627 (0.134)	0.627 (0.137)	0.224	[− 0.012%, 0.052%]
Grade	6.210 (1.764)	6.674 (1.641)	< 0.001	[0.459, 0.467]
CAG	–	6.622	–	–

Standard deviations in parentheses. *p* values derived using Student’s *t*-tests. Treatment grade value is not the same as mean CAG value but represents mean final grades for 2020. See footnote 3 for detail

**Table 5:** Categorical variables—checking for balance

Variable	Category	Control proportion (%)	Treatment proportion (%)	*p* value
EAL	No EAL	85.86	85.00	< 0.001
	EAL	14.14	15.00	< 0.001
Gender	Female	52.08	52.85	< 0.001
	Male	47.92	47.15	< 0.001
Ethnicity	Any Other Ethnic Group	1.48	1.66	< 0.001
	Asian	12.25	13.21	< 0.001
	Black	4.19	4.62	< 0.001
	Chinese	0.71	0.71	< 0.001
	Mixed	5.01	5.54	< 0.001
	White	76.36	74.26	< 0.001
FSM	No FSM	94.45	93.26	< 0.001
	FSM	5.55	6.74	< 0.001
SEN	No SEN	95.80	95.42	< 0.001
	SEN	4.20	4.58	< 0.001
Tier	Foundation	6.04	6.68	< 0.001
	Higher	93.96	93.32	< 0.001
Centre Type	Academies	59.64	61.04	< 0.001
	Free schools	1.22	1.40	< 0.001
	Independent school including city training colleges (CTCs)	0.46	0.44	< 0.001
	Other	0.68	0.71	< 0.001
	Secondary comprehensive or middle school	30.53	28.63	< 0.001
	Secondary modern school/high school	1.20	1.06	< 0.001
	Secondary selective school	6.24	6.70	< 0.001
	Sixth form college	0.0	0.01	< 0.001
	Tertiary college	0.01	0.01	< 0.001

*p* values derived using Chi-squared test. Some centre types omitted due to disclosive (< 10 observations) nature

### Main effects

As Table 6 shows, the CATEs across all main categories were positive and statistically significant at the *p < *0.001 level. The largest effect overall was for very high IDACI students, who received 4.301 more GCSE total grade points than they were modelled to receive. The smallest CATE overall was for students with very high prior attainment (3.015). This gave an inter-group range of 1.286-grade points between the maximum and minimum CATEs.

**Table 6:** CATE | main effects

Variable	Category	CATE	Avg. CAG score	Avg. modelled score	*p* value
SEN	SEN	3.099	51.766	48.667	< 0.001
	No SEN	3.723	59.071	55.347	< 0.001
Prior attainment	Very high	3.015	70.327	67.313	< 0.001
	High	3.552	64.148	60.596	< 0.001
	Medium	3.915	59.681	55.766	< 0.001
	Very low	3.916	45.752	41.836	< 0.001
	Low	4.041	54.625	50.584	< 0.001
IDACI quantile	Very low IDACI	3.204	61.68	58.476	< 0.001
	Low IDACI	3.413	59.847	56.435	< 0.001
	Medium IDACI	3.633	58.629	54.995	< 0.001
	High IDACI	3.921	57.551	53.631	< 0.001
	Very high IDACI	4.301	55.942	51.641	< 0.001
Gender	M	3.493	57.114	53.621	< 0.001
	F	3.876	60.189	56.313	< 0.001
FSM	No FSM	3.687	59.222	55.535	< 0.001
	FSM	3.795	52.074	48.279	< 0.001
Ethnicity	AOEG	3.173	60.401	57.228	< 0.001
	Mixed	3.24	59.907	56.667	< 0.001
	Asian	3.375	61.209	57.834	< 0.001
	Black	3.632	57.115	53.483	< 0.001
	White	3.799	58.183	54.384	< 0.001
	Chinese	3.844	67.782	63.938	< 0.001
EAL	EAL	3.607	60.029	56.422	< 0.001
	No EAL	3.71	58.503	54.793	< 0.001

*p* values derived using Welch’s *t* -tests to assess if differences between modelled scores and CAG scores were statistically significant. Welch’s *t* tests were used as it cannot be assumed these groups have equal variances. Results have been sorted reverse-alphabetically on Variable and then ascendingly on CATE

Within the IDACI category, the order of CATEs ascended identically with the ordinal scale of the variable itself—with very low IDACI students receiving the smallest CATE, low IDACI receiving the next smallest, etc. Within the other quantile variable, prior attainment, CATEs did not ascend identically with the ordinal scale of the variable itself, however. Very high and high prior attainment categories had the smallest CATEs, but the largest CATE was observed for low prior attainment (4.041)—not very low prior attainment. Furthermore, the top three attainment quantiles were much closer together (0.126 between medium and low) versus a 0.537-point jump from very high to high and a 0.363-point jump from high to medium.

**Fig. 3:**
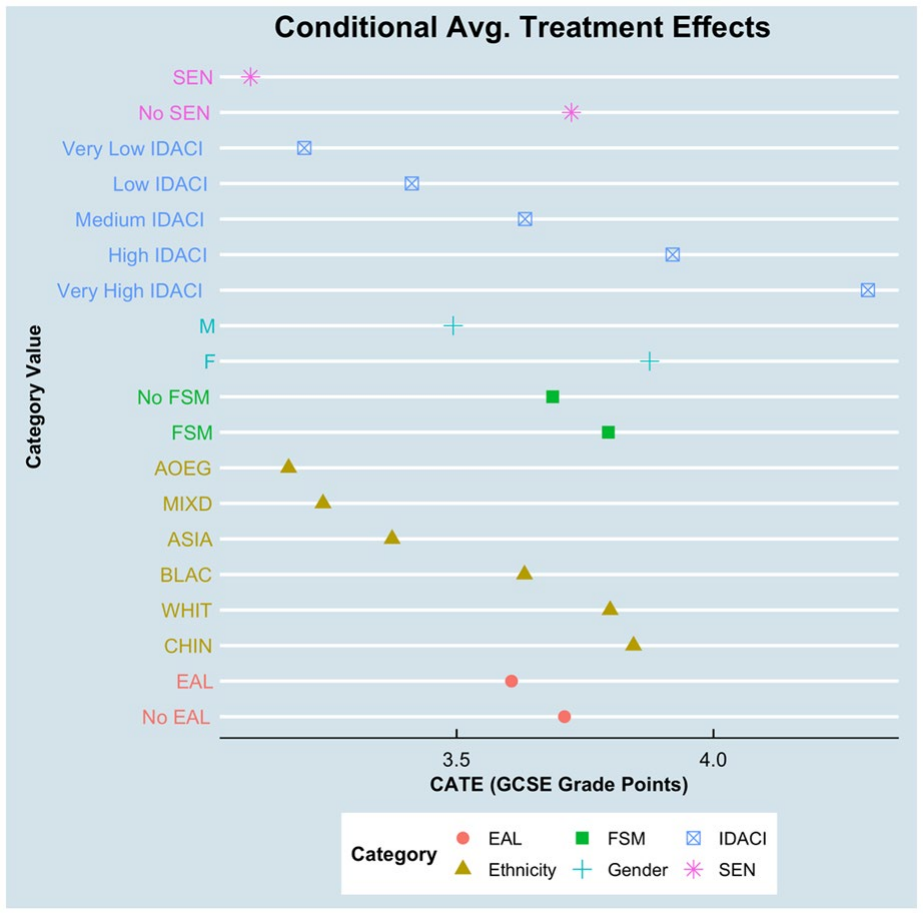
Main effects

The Any Other Ethnic Group (AOEG) students received the smallest ethnicity CATE of 3.173, compared to White and Chinese students who had the two largest CATEs (3.799 and 3.844 respectively). The comparison of ethnicity CATEs is best demonstrated in Fig. 3. Across binary variables, CATEs were 0.624 points larger for No SEN students rather than SEN, 0.383 larger for females rather than males, 0.108 larger for FSM rather than no FSM, and 0.103 larger for no EAL rather than EAL.

The remaining intra-group ranges were 1.026 for prior attainment, 1.097 for IDACI and 0.671 for ethnicity. Intra-group range was therefore smallest for the EAL variable and largest for the IDACI variable.

### Two-way interactions

#### IDACI X prior attainment

**Fig. 4:**
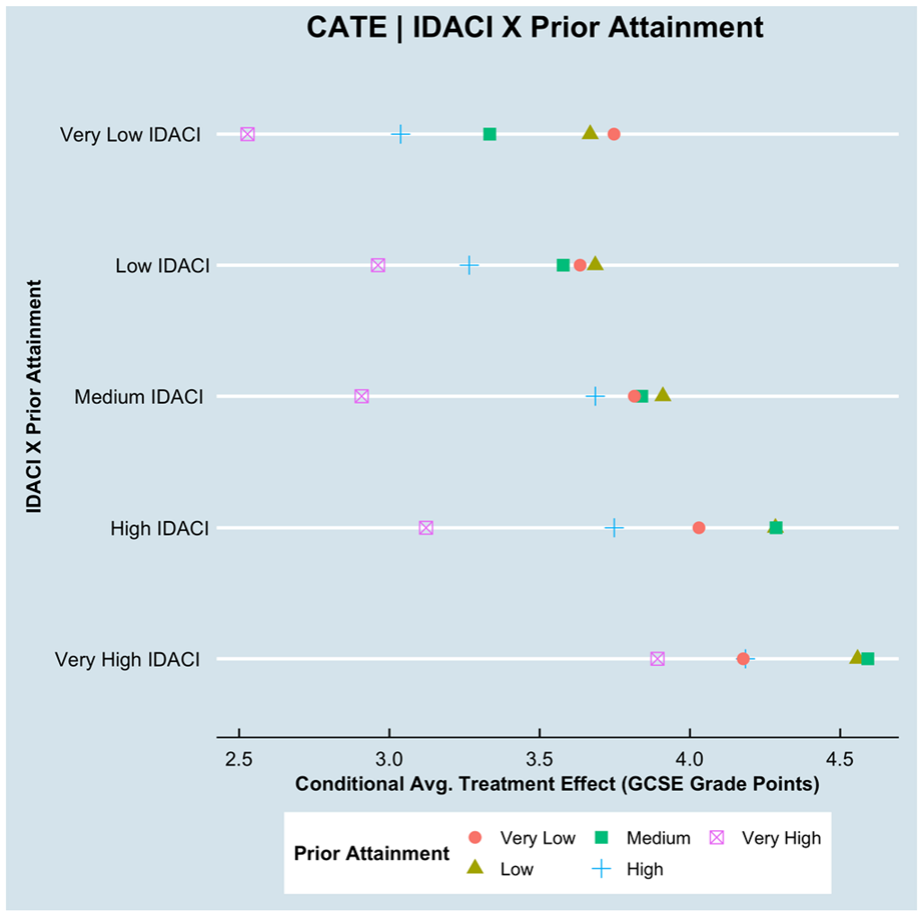
IDACI X Prior Attainment

CATEs were positive and statistically significant at the *p < *0.001 level for all categories of IDACI x prior attainment (see Table 7). The largest CATE (4.592) was for very high IDACI, medium prior attainment students and the smallest CATE (2.528) for very low IDACI, very high prior attainment students. This gave an intra-group range of 2.064-grade points. As Fig. 4 shows, within all IDACI quantiles, students with very high prior attainment received the smallest CATEs. Furthermore, the very high IDACI quantile was the only one in which the second-smallest CATE was not for high prior attainment—with very low prior attaining students having a slightly smaller CATE than high prior attainment students (4.177 vs 4.185).

**Table 7:** CATE | IDACI X prior attainment

Variable	Category	CATE	Avg. CAG score	Avg. modelled score	*p* value
IDACI X prior attainment	Very low idaci x very low	3.747	47.584	43.836	< 0.001
	Very low IDACI X very high	2.528	71.619	69.091	< 0.001
	Very low IDACI X medium	3.334	61.249	57.915	< 0.001
	Very low IDACI X low	3.668	56.357	52.689	< 0.001
	Very low IDACI X high	3.037	65.633	62.596	< 0.001
	Very high IDACI X very Low	4.177	45.103	40.926	< 0.001
	Very high IDACI X very high	3.892	68.494	64.602	< 0.001
	Very high IDACI X medium	4.592	58.268	53.676	< 0.001
	Very high IDACI X low	4.558	53.599	49.041	< 0.001
	Very high IDACI X high	4.185	62.529	58.345	< 0.001
	Medium IDACI X very low	3.815	45.369	41.554	< 0.001
	Medium IDACI X very High	2.908	70.097	67.19	< 0.001
	Medium IDACI X medium	3.84	59.461	55.621	< 0.001
	Medium IDACI X low	3.91	54.411	50.501	< 0.001
	Medium IDACI X high	3.685	64.124	60.439	< 0.001
	Low IDACI X very low	3.634	46.392	42.757	< 0.001
	Low IDACI X very high	2.962	71.017	68.055	< 0.001
	Low IDACI X medium	3.578	60.196	56.618	< 0.001
	Low IDACI X low	3.685	54.806	51.121	< 0.001
	Low IDACI X high	3.266	64.495	61.229	< 0.001
	High IDACI X very low	4.03	45.132	41.102	< 0.001
	High IDACI X very high	3.122	69.541	66.419	< 0.001
	High IDACI X medium	4.287	59.089	54.803	< 0.001
	High IDACI X low	4.285	54.181	49.897	< 0.001
	High IDACI X high	3.748	63.539	59.79	< 0.001

Same notes as Table 6, except results have been sorted reverse-alphabetically on category

Within quantiles of prior attainment, CATEs increase mostly in the same order as the IDACI quantiles, with very low IDACI students having the smallest CATEs and very high IDACI students having the largest CATEs, for any given level of prior attainment. One exception to this is very low prior attaining students whose CATE in the very low IDACI quantile is 3.747 and decreases slightly (by 0.079 points) moving to the low IDACI quantile.

#### IDACI X ethnicity

Table 8 shows that CATEs were positive across all categories of IDACI x ethnicity. Certain observations from AOEG, Black and Chinese sub-groups were not statistically significant, however, and several more observations in those sub-groups were only significant at a lower threshold (*p < *0.05 rather than *p < *0.001). The smallest and largest statistically significant CATEs were 2.427 and 5.137 (a range of 2.710) for very low IDACI, mixed ethnicity students and very high IDACI, Chinese students, respectively.

**Table 8:** CATE | ethnicity X IDACI

Variable	Category	CATE	Avg. CAG score	Avg. modelled score	*p* value
Ethnicity X IDACI	White X very low IDACI	3.299	61.321	58.022	< 0.001
	White X very high IDACI	4.633	53.701	49.068	< 0.001
	White X medium IDACI	3.787	57.851	54.064	< 0.001
	White X low IDACI	3.516	59.274	55.758	< 0.001
	White X high IDACI	4.207	56.402	52.195	< 0.001
	Mixed X very low IDACI	2.427	62.569	60.142	< 0.001
	Mixed X very high IDACI	3.739	57.456	53.717	< 0.001
	Mixed X medium IDACI	2.961	60.424	57.463	< 0.001
	Mixed X low IDACI	3.068	61.96	58.892	< 0.001
	Mixed X high IDACI	3.581	59.065	55.484	< 0.001
	Chinese X very low IDACI	2.891	69.138	66.248	0.0641
	Chinese X very high IDACI	5.137	66.985	61.849	< 0.001
	Chinese X medium IDACI	3.946	67.687	63.741	0.0104
	Chinese X low IDACI	3.204	67.793	64.589	0.0322
	Chinese X high IDACI	3.489	67.784	64.295	0.0063
	Black X very low IDACI	3.204	61.034	57.83	0.0269
	Black X very high IDACI	4.047	56.591	52.544	< 0.001
	Black X medium IDACI	2.895	59.204	56.309	< 0.001
	Black X low IDACI	1.66	57.482	55.823	0.1293
	Black X high IDACI	3.305	57.078	53.772	< 0.001
	Asian X very low IDACI	2.436	65.841	63.406	< 0.001
	Asian X very high IDACI	3.98	59.029	55.049	< 0.001
	Asian X medium IDACI	3.137	62.8	59.663	< 0.001
	Asian X low IDACI	2.793	64.898	62.105	< 0.001
	Asian X high IDACI	3.256	60.349	57.093	< 0.001
	AOEG X very low IDACI	1.927	64.425	62.498	0.2332
	AOEG X very high IDACI	3.66	59.228	55.568	< 0.001
	AOEG X medium IDACI	1.5	60.324	58.825	0.1929
	AOEG X low IDACI	2.334	64.117	61.783	0.0848
	AOEG X high IDACI	3.783	60.284	56.5	< 0.001

Same notes as Table 7

**Fig. 5:**
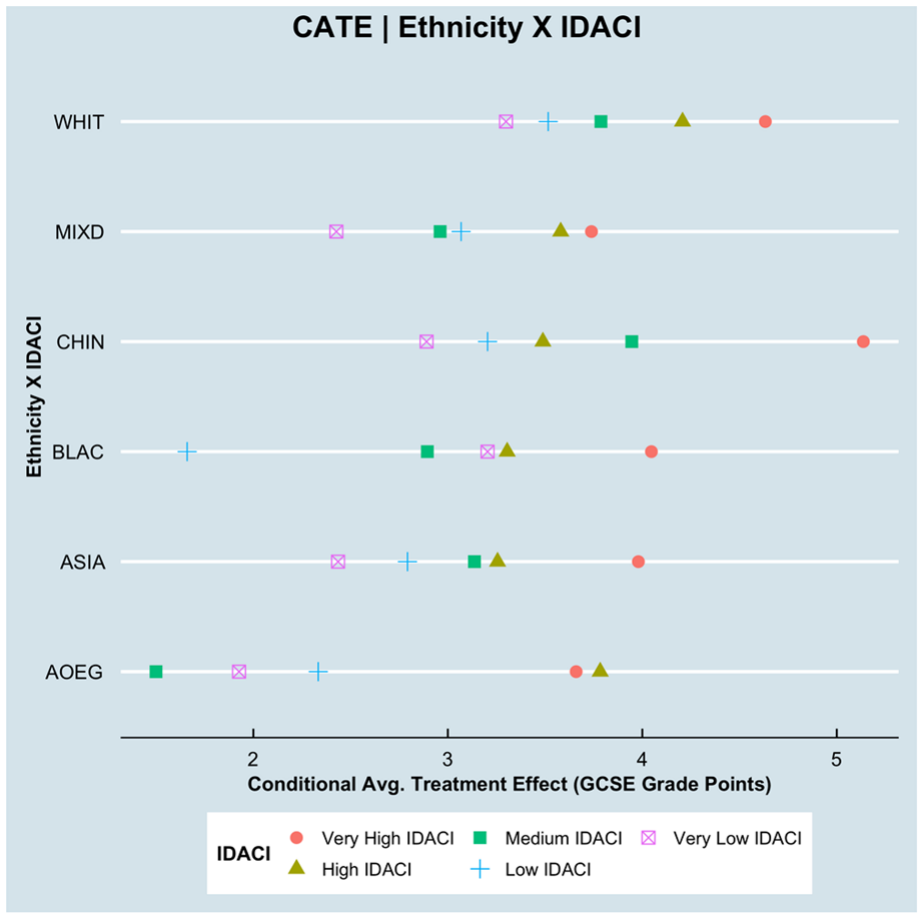
Ethnicity X IDACI

CATEs within ethnicities generally increased in the same order as the IDACI quantiles, with the smallest CATEs being observed for very low IDACI students and the largest CATEs for very high IDACI students. However, these orders were not perfectly followed in the groups that contained results that were not statistically significant. Figure 5 shows that when the order of CATEs does not follow that of the IDACI quantiles, it is results that are not statistically significant or are significant at a lower level that break it.

#### Ethnicity X prior attainment

Table 9 shows that CATEs were positive across all categories of ethnicity x prior attainment. They were all also statistically significant, though some results for AOEG and Chinese students were significant at lower levels (*p < *0.05). The largest CATE was for Chinese students with very low prior attainment (5.240) and the smallest CATE was for AOEG students with very high prior attainment (1.614), with a difference between them of 3.626-grade points.

**Table 9:** CATE | ethnicity X prior attainment

Variable	Category	CATE	Avg. CAG score	Avg. modelled score	*p* value
Ethnicity X prior attainment	White X very low	3.929	44.562	40.633	< 0.001
	White X very high	3.19	69.815	66.625	< 0.001
	White X medium	4.033	58.921	54.888	< 0.001
	White X low	4.177	53.766	49.588	< 0.001
	White X high	3.662	63.493	59.83	< 0.001
	Mixed X very low	3.562	46.229	42.667	< 0.001
	Mixed X very high	2.58	71.504	68.924	< 0.001
	Mixed X medium	3.547	60.889	57.341	< 0.001
	Mixed X low	3.532	55.604	52.072	< 0.001
	Mixed X high	3.012	65.14	62.128	< 0.001
	Chinese X very low	5.24	54.83	49.59	< 0.001
	Chinese X very high	2.477	76.526	74.05	0.0061
	Chinese X medium	4.601	66.211	61.61	< 0.001
	Chinese X low	3.942	61.008	57.066	0.0016
	Chinese X high	4.108	70.006	65.898	< 0.001
	Black X very low	3.938	46.75	42.812	< 0.001
	Black X very high	3.062	69.897	66.834	< 0.001
	Black X medium	3.574	60.266	56.692	< 0.001
	Black X low	3.484	55.549	52.064	< 0.001
	Black X high	3.79	65.013	61.223	< 0.001
	Asian X very low	3.887	49.726	45.84	< 0.001
	Asian X very high	2.329	72.331	70.002	< 0.001
	Asian X medium	3.643	62.927	59.285	< 0.001
	Asian X low	3.848	57.798	53.949	< 0.001
	Asian X high	2.954	66.793	63.839	< 0.001
	AOEG X very low	4.238	50.594	46.356	< 0.001
	AOEG X very high	1.614	71.678	70.064	0.0489
	AOEG X medium	2.229	61.883	59.654	0.0016
	AOEG X low	3.058	58.455	55.396	< 0.001
	AOEG X high	4.128	67.14	63.012	< 0.001

Same notes as Table 7

**Fig. 6:**
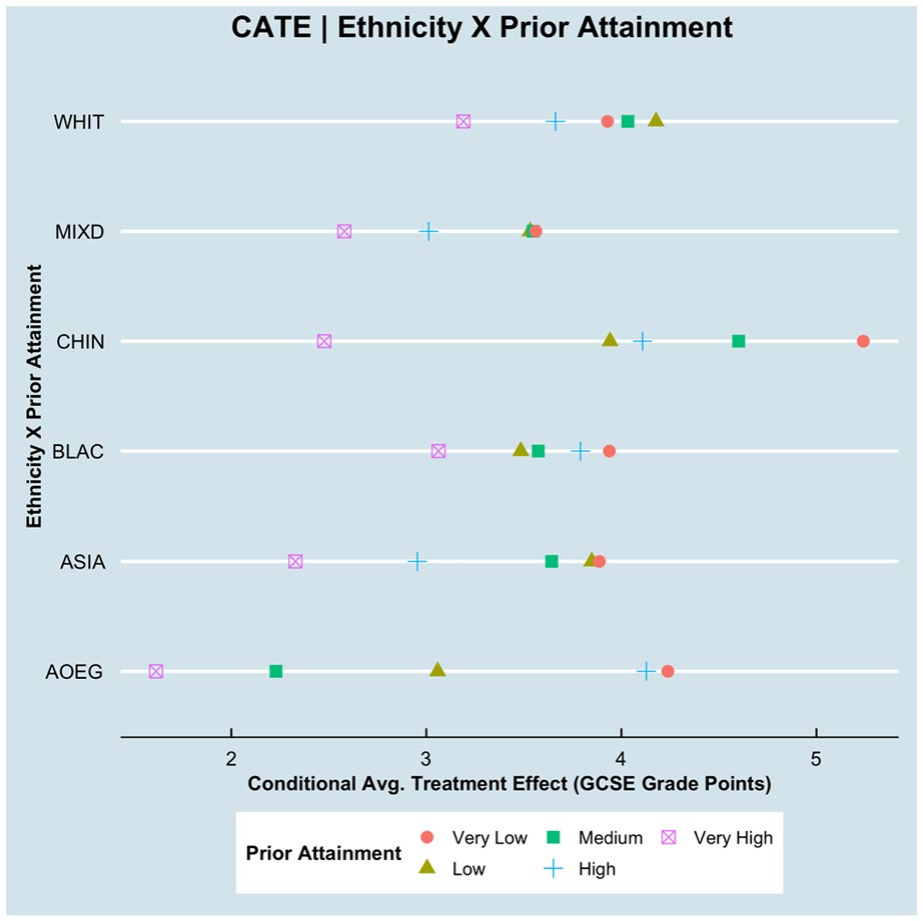
Ethnicity X prior attainment

Within ethnicities, students with very high prior attainment received the smallest CATEs and students with very low prior attainment generally received the largest CATEs (see Fig. 6). An exception to this is the White sub-group, where medium (4.033) and low (4.177) prior attainment students had larger CATEs than very low (3.929) prior attainment students. The ranges of CATEs across attainment levels differ by ethnicity as well. Chinese and AOEG students have the largest differences between their maximum and minimum attainment quantile CATEs, with differences of 2.763 and 2.624-grade points respectively. In contrast, White, Black, and Mixed ethnicity students have narrower ranges of 0.987, 0.876 and 0.982-grade points, respectively.

Within prior attainment quantiles, the AOEG group received the smallest CATEs for very high, medium, and low quantiles. White students received the largest CATEs for very high, high, and low quantiles of prior attainment. Chinese students received the largest CATEs for medium and very low quantiles.

#### IDACI X SEN

CATEs for all categories of IDACI x SEN were positive and statistically significant, as Table 10 shows. The range between the largest (no SEN students with very high IDACI, 4.344) and smallest CATEs (SEN students with low IDACI, 1.996) was 2.348-grade points.

**Table 10:** CATE | IDACI X SEN

Variable	Category	CATE	Avg. CAG score	Avg. modelled score	*p* value
IDACI X SEN	SEN X very low IDACI	3.048	55.863	52.815	< 0.001
	SEN X very high IDACI	3.632	48.271	44.639	< 0.001
	SEN X medium IDACI	3.365	52.361	48.997	< 0.001
	SEN X low IDACI	1.996	52.604	50.608	0.001
	SEN X high IDACI	3.39	49.911	46.521	< 0.001
	No SEN X very low IDACI	3.211	61.959	58.748	< 0.001
	No SEN X very high IDACI	4.334	56.328	51.994	< 0.001
	No SEN X medium IDACI	3.646	58.935	55.289	< 0.001
	No SEN X low IDACI	3.48	60.19	56.71	< 0.001
	No SEN X high IDACI	3.947	57.925	53.978	< 0.001

Same notes as Table 7

**Fig. 7:**
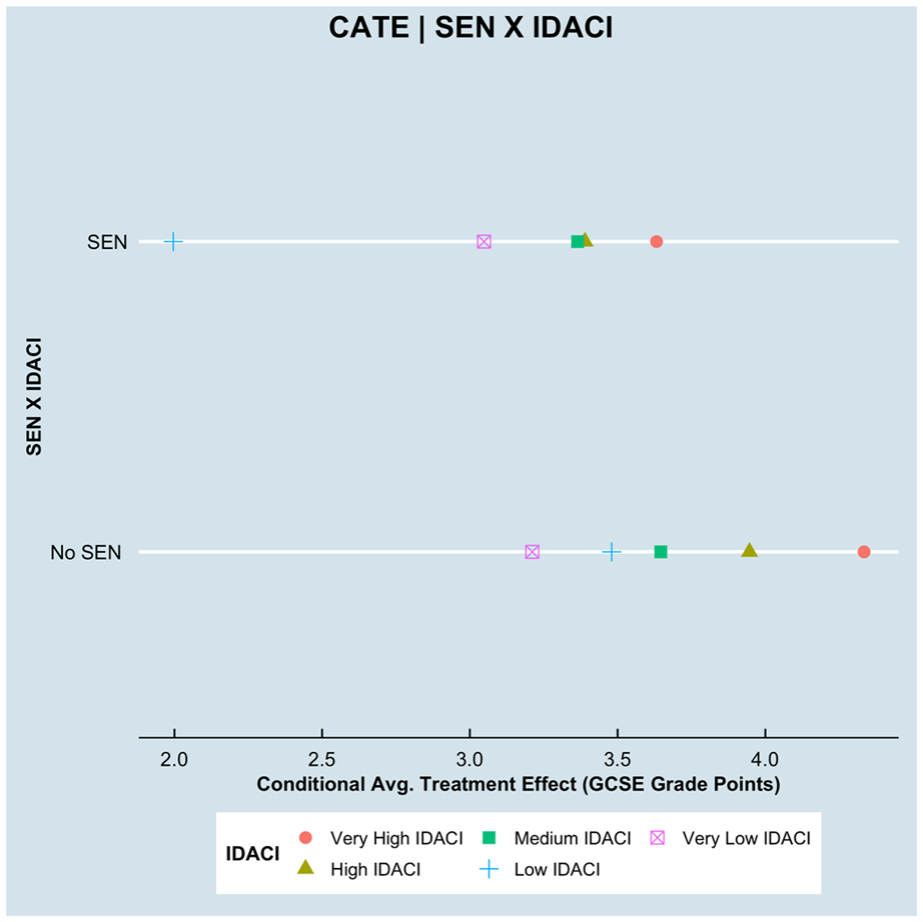
IDACI X SEN

As Fig. 7 shows, within IDACI quantiles, no SEN students received larger CATEs than SEN students. However, these no SEN increases were largest for the low and very high IDACI quantiles (1.484 and 0.702 increases respectively). Within SEN categories, CATEs generally increased in the same order as the IDACI quantiles, from very low (smallest) to very high (largest). However, the SEN category had low IDACI with the smallest CATE and very low IDACI as the second smallest.

#### Prior attainment X SEN

As Table 11 shows, CATEs for all categories of prior attainment x SEN were positive and statistically significant. SEN students with medium prior attainment received the lowest CATE (2.524) though this was almost identical to the CATE of very high-attaining SEN students (2.523). SEN students with low prior attainment had the largest CATE (4.063), giving a range of 1.540-grade points.

**Table 11:** CATE | prior attainment X SEN

Variable	Category	CATE	Avg. CAG score	Avg. modelled score	*p* value
Prior Attainment X SEN	SEN X very low	3.235	40.586	37.351	< 0.001
	SEN X very high	2.523	66.532	64.009	< 0.001
	SEN X medium	2.524	55.895	53.371	< 0.001
	SEN X low	3.586	52.08	48.494	< 0.001
	SEN X high	3.269	61.403	58.134	< 0.001
	No SEN X very low	3.977	46.209	42.233	< 0.001
	No SEN X very high	3.03	70.444	67.414	< 0.001
	No SEN X medium	3.972	59.835	55.864	< 0.001
	No SEN X low	4.063	54.745	50.683	< 0.001
	No SEN X high	3.563	64.247	60.685	< 0.001

Same notes as Table 7

**Fig. 8:**
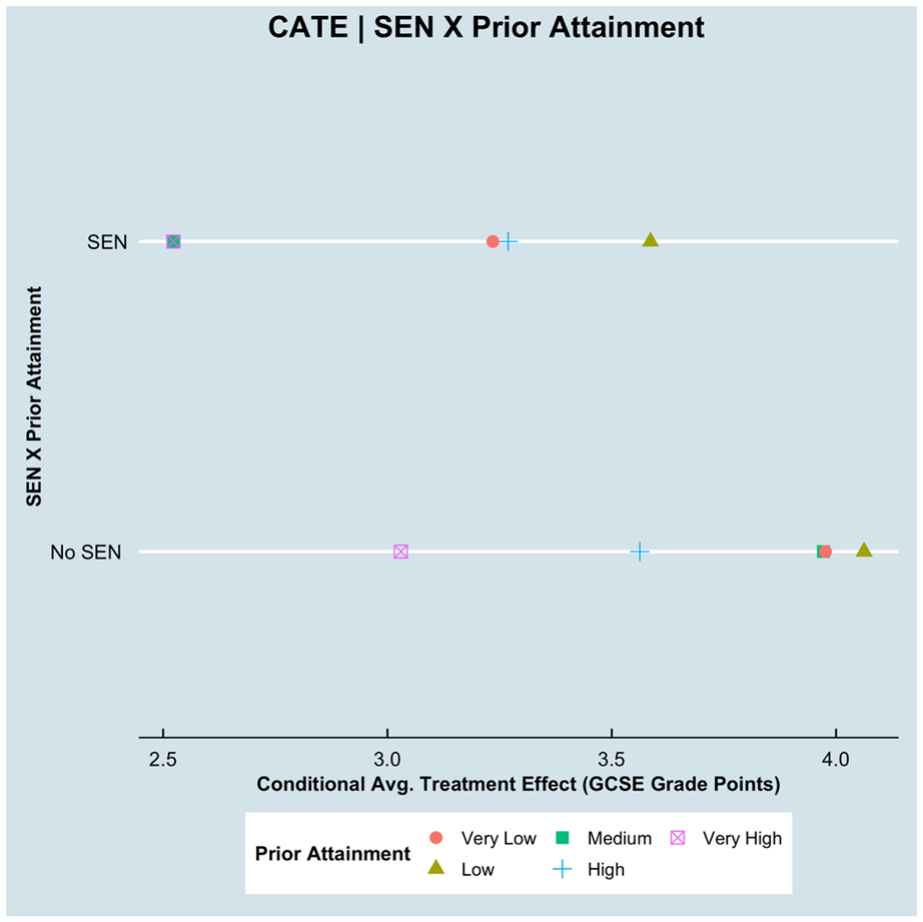
Prior attainment X SEN

Within all attainment quantiles, no SEN students received larger CATEs than SEN students (see Fig. 8). Within SEN categories, the order of increasing CATEs does not follow the order of increasing attainment quantiles. Low-attaining students have the largest CATEs across both SEN categories and very high-attaining students have (virtually, in SEN’s case) have the lowest CATEs.

#### IDACI X gender

Positive, statistically significant CATEs were observed for all categories of IDACI x gender, as Table 12 demonstrates. The largest CATE was for very high IDACI, female students with 4.340-grade points. The lowest CATE was for very low IDACI, male students (2.988). This gave an intra-group range of 1.352-grade points. Within both genders, CATEs increased by IDACI quantile in the exact same order as the IDACI quantiles themselves, i.e., very low (smallest) to very high (largest).

**Table 12:** CATE | IDACI X gender

Variable	Category	CATE	Avg. CAG score	Avg. modelled score	*p* value
IDACI X Gender	M X very low IDACI	2.988	59.831	56.843	< 0.001
	M X very high IDACI	4.252	54.574	50.322	< 0.001
	M X medium IDACI	3.41	56.934	53.524	< 0.001
	M X low IDACI	3.183	58.058	54.875	< 0.001
	M X high IDACI	3.685	55.971	52.285	< 0.001
	F X very low IDACI	3.406	63.41	60.004	< 0.001
	F X very high IDACI	4.34	57.06	52.72	< 0.001
	F X medium IDACI	3.838	60.184	56.345	< 0.001
	F X low IDACI	3.626	61.513	57.886	< 0.001
	F X high IDACI	4.135	58.989	54.854	< 0.001

Same notes as Table 7

**Fig. 9:**
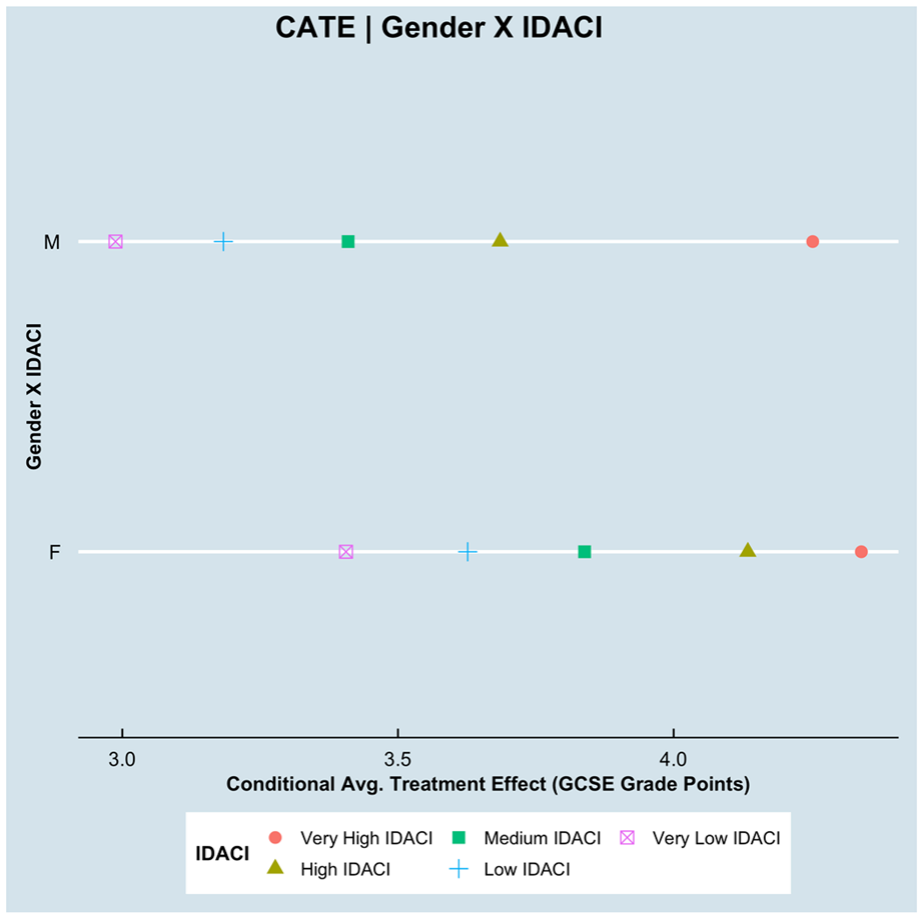
IDACI X gender

As Fig. 9 shows, within each given IDACI quantile, female students received larger CATEs. These respective increases in female students were similar in size except for the very high IDACI quantile, which only saw an increase of 0.088 grade points (4.252 males to 4.340 females).

#### Prior attainment X gender

CATEs for all categories of prior attainment x gender were positive and statistically significant (see Table 13). Female students with medium prior attainment had the largest CATE (4.200) and male students with very high attainment had the smallest CATE (2.924). This produced a range of 1.276-grade points.

**Table 13:** CATE | prior attainment X gender

Variable	Category	CATE	Avg. CAG score	Avg. modelled score	*P* value
Prior attainment X gender	M X very low	3.626	43.892	40.266	< 0.001
	M X very high	2.924	68.347	65.422	< 0.001
	M X medium	3.603	57.302	53.699	< 0.001
	m x low	3.934	52.301	48.367	< 0.001
	M X high	3.426	61.983	58.557	< 0.001
	F X very low	4.132	47.135	43.003	< 0.001
	F X very high	3.109	72.396	69.287	< 0.001
	F X medium	4.2	61.854	57.654	< 0.001
	F X low	4.132	56.602	52.47	< 0.001
	F X high	3.678	66.302	62.624	< 0.001

Same notes as Table 7

**Fig. 10:**
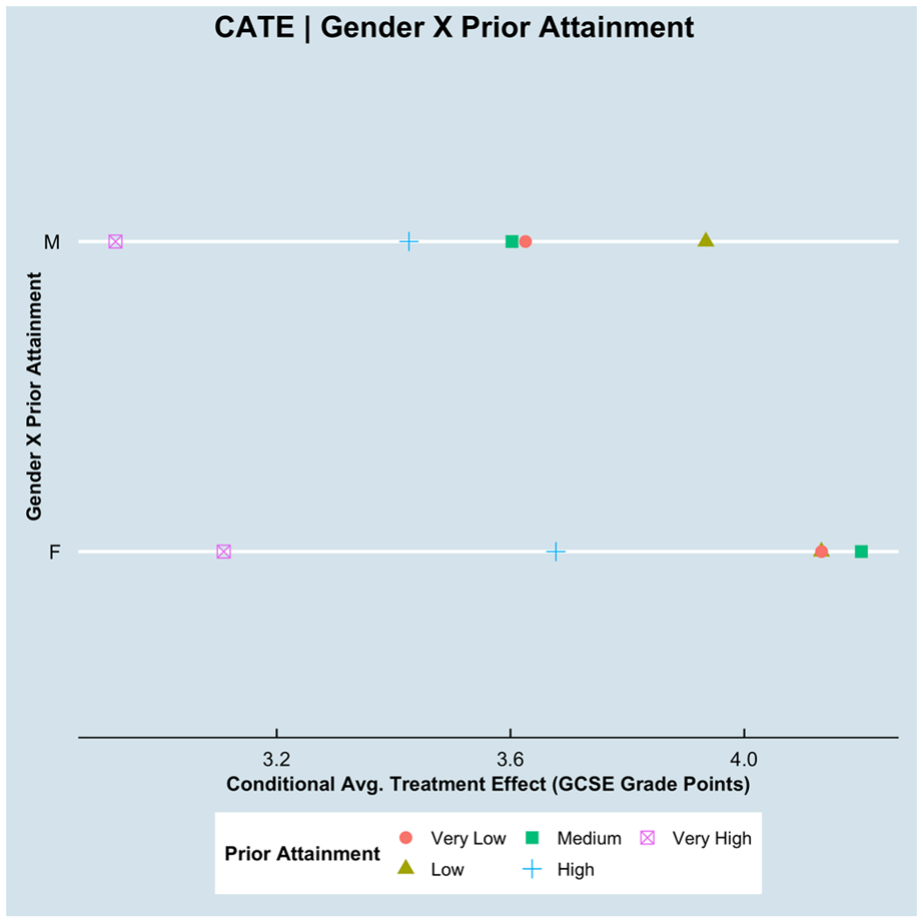
Prior attainment X gender

As Fig. 10 shows, within all attainment quantiles, female students received larger CATEs than male students. Within gender categories, the order of increasing CATEs does not follow the order of increasing attainment quantiles. Very high and high prior-attaining students have the lowest CATEs respectively. For males, students with low attainment have the largest CATE, followed by very low attainment students (a difference of 0.308-grade points). In contrast, for females it is the medium attainment students who have the largest CATE. However, the gap to the next largest quantile (which is a tie between low and very low attainment) is smaller than the gap is in men’s—with only a 0.068-grade point difference.

### Three-way interactions

#### Ethnicity X IDACI X prior attainment

Positive CATEs were observed across all categories of ethnicity x IDACI x prior attainment. Not all were statistically significant, and many were only significant at higher thresholds (*p < *0.05), however (see Table 14). The largest CATE (5.153) was for White, very high IDACI students with low prior attainment. Indeed, 6 out of 10 of the top 10 largest CATEs belonged to White students with either high or very high IDACI quantiles. The smallest CATE (1.382) was for Asian, very low IDACI students with very high prior attainment. This gave an intra-group range of 3.771-grade points between the largest and smallest observed CATEs.

**Table 14:** CATE | ethnicity X IDACI X prior attainment

Variable	Category	CATE	Avg. CAG score	Avg. modelled score	*p* value
Ethnicity X IDACI X prior attainment	White X very high IDACI X low	5.153	51.175	46.022	< 0.001
	White X very high IDACI X medium	5.048	55.685	50.636	< 0.001
	AOEG X very high IDACI X very low	4.929	50.73	45.801	< 0.001
	AOEG X high IDACI X low	4.692	60.218	55.526	< 0.001
	Mixed X high IDACI X medium	4.661	60.951	56.29	< 0.001
	White X high IDACI X medium	4.605	57.771	53.166	< 0.001
	Asian X very high IDACI X medium	4.598	61.983	57.385	< 0.001
	White X very high IDACI X High	4.587	60.481	55.894	< 0.001
	White X high IDACI X Low	4.541	52.605	48.064	< 0.001
	Asian X medium IDACI X very low	4.487	51.077	46.59	< 0.001
	Asian X very low IDACI X high	2.129	69.502	67.373	0.0067
	Asian X low IDACI X very high	2.109	73.841	71.732	0.0015
	Mixed X medium IDACI X low	2.03	54.857	52.827	0.0209
	Mixed X very low IDACI X high	1.919	65.768	63.849	0.0247
	Mixed X high IDACI X very high	1.783	69.495	67.711	0.0395
	Mixed X very low IDACI X very high	1.725	73.435	71.71	0.0531
	Asian X very low IDACI X medium	1.633	64.298	62.665	0.0374
	Asian X medium IDACI X very high	1.534	72.725	71.192	0.0065
	Black X high IDACI X very high	1.383	69.255	67.872	0.2348
	Asian X very low IDACI X very high	1.382	74.277	72.895	0.0457

Results are truncated here – only the top 10 (white) and bottom 10 (grey) categories in terms of CATE are shown. Results have been sorted descendingly on CATE. See Table 6 for other notes

Across ethnicities and within IDACI quantiles, CATEs were generally the smallest for students with very high or high prior attainment. Black with very high IDACI and Mixed students with very high and medium IDACI were exceptions to this, however, with low, low, and medium-attaining students having the smallest CATEs in those categories.

**Fig. 11:**
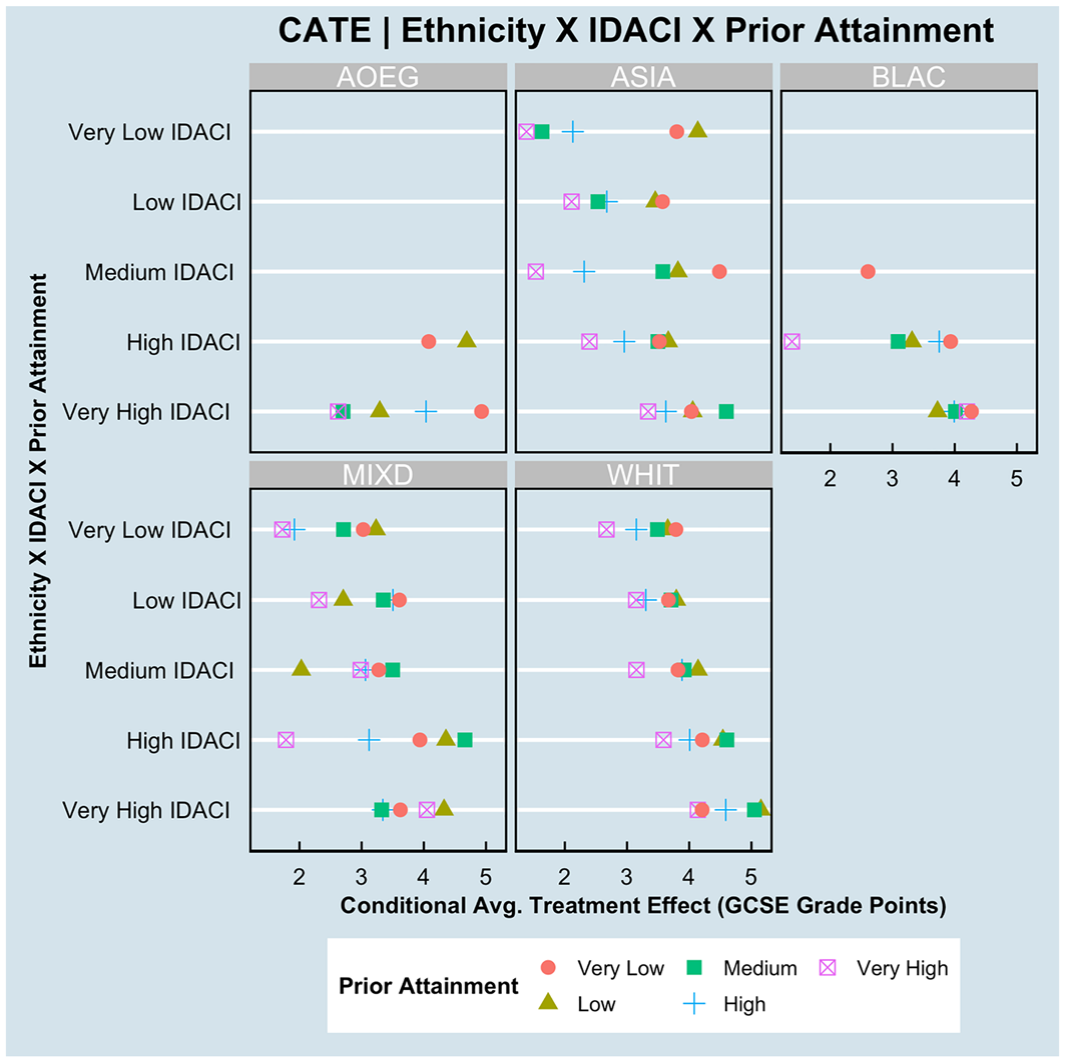
Ethnicity X IDACI X prior attainment

Full results could not be shared for all ethnic groups^9^ but certain patterns emerged in the 3 ethnic groups that did have full results (Asian, Mixed and White). As Fig. 11 highlights, CATEs were more variable for Asian and Mixed students than White students, when looking within both attainment and IDACI quantiles. Furthermore, across all three of these ethnicities and within attainment quantiles, CATEs generally increase in the same order as the IDACI quantiles themselves, e.g., White, very high attaining students have a CATE of 2.670 in the very low IDACI quantile which increases by 1.116-grade points to a maximum of 3.786 for equivalent students in the very high IDACI quantile. An exception to this CATE increase with IDACI across all three ethnicities, however, is for students in the very low attainment quantiles. Mixed, White, and Asian students with very low attainment saw comparatively little increase in CATE as IDACI increased. For example, very low attaining White students in the very low IDACI quantile had a CATE of 3.786, which only increased by 0.421-grade points to 4.207 for equivalent students in the very high IDACI quantile. This was only 37.7% of the CATE increase that equivalent students with very high prior attainment received when moving between the same IDACI quantiles.

#### IDACI X prior attainment X gender

All CATEs across all categories of IDACI x prior attainment x gender were positive and significant at the *p < *0.001 level. The range between the highest and lowest observed CATEs was 2.467-grade points, from female students with very high IDACI and medium prior attainment (4.819) to male students (2.352) with very low IDACI and very high prior attainment (see Table 15). 7 out of the top 10 largest CATEs belonged to females with either very high or high IDACI. 7 out of 10 of the smallest CATEs belonged to males, 4 of whom had very high prior attainment.

**Table 15:** CATE | IDACI X prior attainment X gender

Variable	Category	CATE	Avg. CAG score	Avg. modelled score	*p* value
IDACI X prior attainment X gender	F X very high IDACI X medium	4.819	60.123	55.304	< 0.001
	M X very high IDACI X low	4.79	51.738	46.948	< 0.001
	F X high IDACI X medium	4.555	61.1	56.544	< 0.001
	F X high IDACI X low	4.468	56.143	51.675	< 0.001
	F X very high IDACI X low	4.372	55.088	50.715	< 0.001
	F X high IDACI X very low	4.324	46.532	42.208	< 0.001
	M X very high IDACI X medium	4.322	56.061	51.739	< 0.001
	F X very high IDACI X very low	4.284	46.297	42.014	< 0.001
	F X Very high IDACI X high	4.187	64.361	60.174	< 0.001
	M X very high IDACI X high	4.183	60.629	56.446	< 0.001
	M X very low IDACI X high	3.136	63.636	60.5	< 0.001
	M X high IDACI X very high	3.105	67.546	64.441	< 0.001
	M X low IDACI X high	3.064	62.223	59.159	< 0.001
	M X medium IDACI X very high	2.97	68.201	65.23	< 0.001
	M X very low IDACI X medium	2.964	58.64	55.676	< 0.001
	F X very low IDACI X high	2.938	67.641	64.703	< 0.001
	F X medium IDACI X very high	2.84	72.143	69.303	< 0.001
	M X low IDACI X very high	2.752	69.041	66.288	< 0.001
	F X very low IDACI X very high	2.717	73.812	71.095	< 0.001
	M X very low IDACI X very high	2.352	69.574	67.222	< 0.001

Same notes as Table 14

**Fig. 12:**
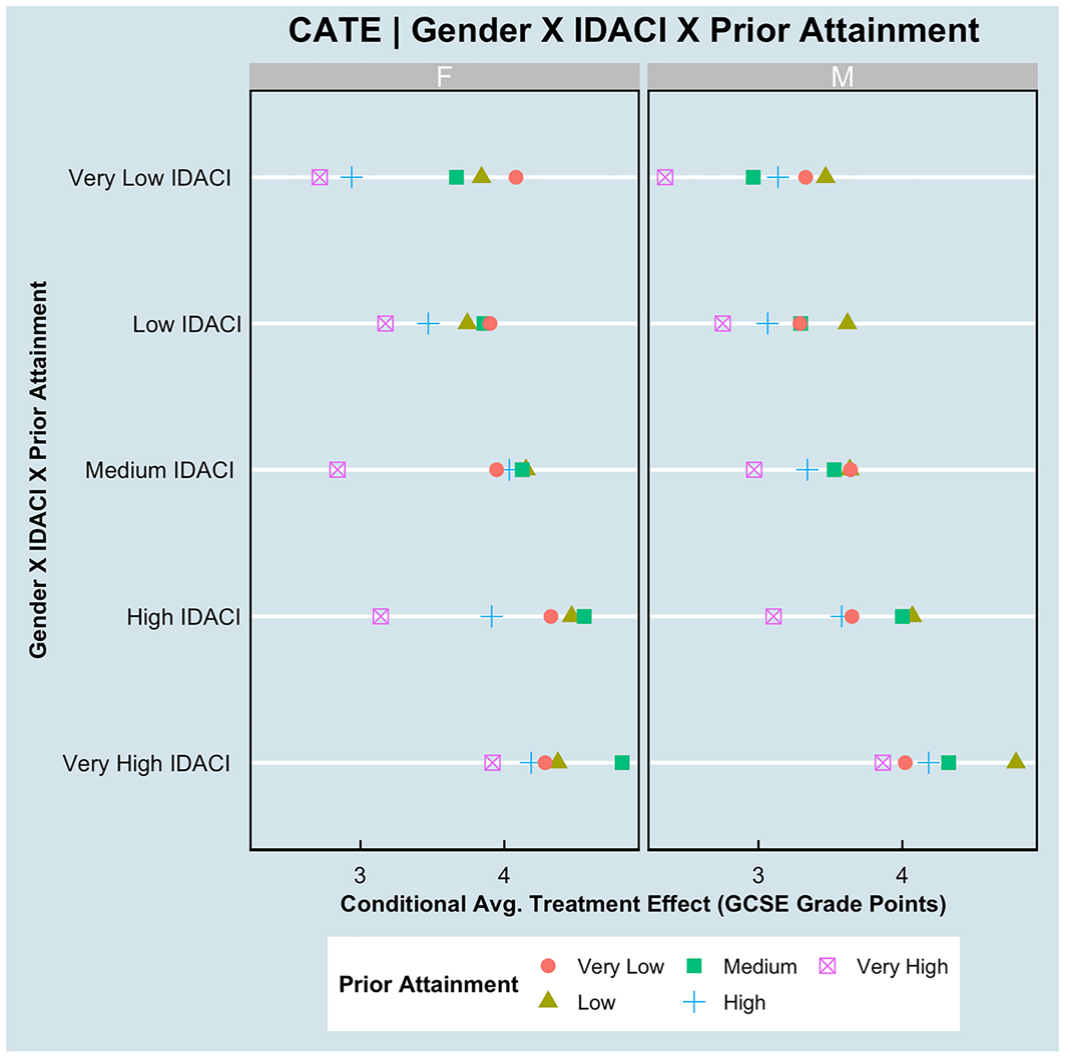
IDACI X prior attainment X gender

As Fig. 12 highlights, within IDACI quantiles and across genders, students with very high or high prior attainment generally have the smallest CATEs. For males, students with low prior attainment received the largest CATEs in all IDACI quantiles (other than the medium IDACI quantile, where very low attainment students have a CATE that was barely (0.004) larger). In contrast, medium-attainment females had the largest CATEs in high and very high IDACI quantiles and very low-attainment females had the largest CATEs in the very low and low IDACI quantiles.

Within IDACI and attainment quantiles, females received larger CATEs in all but 3 out of the 25 possible gender comparisons (see Appendix C). Within genders and attainment quantiles, CATEs generally increased with the order of the IDACI quantiles. However, these increases were the smallest for students with very low or low prior attainment.

## Discussion

### No absolute negative bias

No absolute negative bias in teacher judgements was found in any of the results presented here or in Appendix C. That is to say, it is unlikely that students belonging to any of the combinations of protected characteristics considered here were worse off in terms of their teacher-assessed grades when compared to what they would have been likely to have received had COVID interruptions not occurred and GCSE examinations had gone ahead as normal. This is evidenced by the fact that the CATEs for all groups and sub-groups considered were positive.^10^ This finding aligns with prior research on the use of predicted grades in the UK. Wyness [51] and Shiner and Modood [42] both demonstrated that teachers are more likely to over-predict than under-predict when making A-level predictions for university applications. Over-prediction would seem to have been the case in the CAG process as well. Such a result also concurs with existing research into the CAGs specifically. Both the Ofqual investigations [27, 45] around grading in 2020 concluded that there was no evidence of systematic bias against students in terms of their protected characteristics. Given the potential impacts of the teacher assessments and the scale with which they were used here, it is good to have corroborated those findings. However, given that much of the education system is rivalrous within cohorts (e.g., admission to university), merely ruling out absolute negative bias/under-prediction for 2020 compared to 2018/19 is insufficient. There may not have been students who were disadvantaged by the teacher assessments in terms of their protected characteristics when looking *across* cohorts—but there may still have been students who were relatively disadvantaged when looking *within* their cohort.

### Relative bias

For the teacher assessments used during the CAG process to have been as relatively equitable or inequitable as regular GCSE examinations, the treatment effects of the use of those assessments would be needed to have been the same for all types of students. This was not the case. Although no students were likely to have been under-predicted relative to 2018/19 examinations, the degree of over-prediction varied according to certain protected characteristics of the students. A consistent example of this was demonstrated by the IDACI variable, a proxy for SES. CATEs were found to increase with IDACI (increase as SES lowered) and in the same order as the IDACI quantiles. This was observed in the main effects of IDACI, as well as across categories of ethnicity, EAL, SEN, gender, prior attainment, ethnicity and prior attainment, and gender and prior attainment. These last three interactions are particularly important because as Stratton, Zanini and Noden [45] note, there is a “ceiling effect” on grades such that very high prior attainment students (who are disproportionately high SES) cannot be over-predicted, only accurately or under-predicted. Without considering the interactions of SES with prior attainment, one might think that the decreases in CATEs as SES increased was due to this ceiling effect. However, as this study shows, even among students with very high prior attainment CATEs decreased as SES increased. This result contradicts Murphy and Wyness [33] who find that among high-achieving students in the UK, lower SES students tend to receive slightly lower predicted grades. This contradiction could perhaps be due sample differences – with their study being based on A-Level students, who have a smaller low-SES proportion than there is among GCSE students [41]. Indeed, the only attainment quantile in this study that did not seem to receive larger CATEs as SES decreased was that of very low prior attainment. The CATEs of very low prior attainment students were found to be relatively stable in interactions of IDACI with prior attainment, and of IDACI with prior attainment and ethnicity.

Although there may have been SES differences in the amount of over-prediction students received, it should be stated that many of these differences were not particularly substantial, e.g., barely a single grade point’s difference in CATEs between very low and very high IDACI at the main level. Considering the CATEs have been summed over the total 8 + GCSEs that students took, the effect of this difference in any single GCSE is not likely to have been major. This concurs with the Ofqual investigations of the CAGs [27, 45] which found that while SES main effects may have been statistically significant, their magnitudes were small overall.

Some small differences in CATEs in terms of ethnicity were also observed. At a main level, Chinese and White students were the two most over-predicted ethnic groups. They were also often the two most over-predicted ethnic groups when looking within both prior attainment and IDACI quantiles. This goes against the work of Shiner and Modood [42] who found evidence in the UK that Black and Indian/Pakistani/Bangladeshi students were more over-predicted than Chinese students, who were themselves more over-predicted than White students. While this disagrees with the current study, it should be noted that many of the CATEs across ethnicities here were somewhat inconsistent. Intra-group ranges, though generally small, were highly variable, as were the orders of CATEs within attainment and IDACI quantiles. This inconsistency in ethnic CATE differences could be reflecting complex interactions that ethnicity has, though it may also reflect the variability introduced into the LGBM model’s predictions by the smaller numbers of observations for certain ethnic minority sub-groups. Further analysis with an ethnicity focus would be needed to confirm or refute Shiner and Modood’s findings.

The patterns for the remaining protected characteristics’ impacts on teacher assessments of academic ability were somewhat clearer than that of ethnicity. Small, but consistent biases in favour of no SEN rather than SEN were observed – with no SEN students having larger CATEs within all IDACI and attainment quantiles. This concurs with Harlen [20], who also found a bias in teacher assessments in favour of no SEN students. Gender had a smaller main effect difference between its categories (in favour of females) than SEN did, however consistent effects were noted for it too. In interactions of gender with prior attainment and IDACI, females received larger CATEs than males in each respective quantile. Furthermore, in an interaction with gender and both prior attainment and IDACI together, females received larger CATEs in all but 3 out of the 25 comparisons with their male counterparts. Lee and Walter [28] also find evidence of small gender effects on teacher judgements but note that it is inconsistent across subjects. Given that this study aggregates to a student level, the gender effects reported here may be obscuring more nuanced, subject-level gender effects.

Lee and Walter also found minimal evidence for bias in terms of EAL, which would be the conclusions of this study too. A very small bias in favour of no EAL students was observed, though it had a smaller main effect difference than both SEN and gender. It did display similar patterns to SEN and gender (see Appendix E), however, with a positive bias in favour of no EAL students found across each quantile of both prior attainment and IDACI. Indeed, although the biases in favour of no SEN, female and no EAL students were consistent, none of them were particularly substantial on their own.

### Intersections matter

That the main effect differences across all variables were insubstantial (even if consistent) supports the conclusions of the two Ofqual investigations [27, 45] of the CAGs which did not find evidence of systematic bias. However, neither of these studies use an intersectional perspective and only consider main or lower-order interaction effects. Bearing in mind what is known about the psychology of bias, focussing on predominantly on the main effects may not be the best way to analyse the topic. As Kunda and Thagard [26] state, stereotypes and individuating information are processed simultaneously by the mind—interacting and jointly influencing each other to produce distinct impressions of people. In the context of teacher judgements, it, therefore, seems unlikely that bias would manifest additively according to protected characteristics. The results of this study would seem to support this statement. The largest intra-group range among main effects was 1.097-grade points, the largest among the two-way interactions was 3.626, and the largest among the three-way interactions was 3.771.^11^ A 3.771-grade point difference between the groups of ethnicity x IDACI x prior attainment with the largest and smallest CATEs is not insubstantial. Even spread over 8 + GCSEs (0.471-grade points per subject if 8 GCSEs taken), which have discrete grade units, it could potentially be a grade’s difference in each subject (with rounding up or down) between the sub-groups with the largest and smallest CATEs. This finding must be contextualised within the methods used to produce it though. The LGBM model’s CATE estimates are likely to have become more variable as the order of interaction increases since higher-order sub-groups will have fewer observations to average over. Nevertheless, even if the main effects of protected characteristics are small, as Ofqual [27, 45] have noted, it would seem they can compound and interact in complex ways to produce effects that are of some substantive importance. Teacher assessments would therefore appear to be susceptible to bias according to certain intersections of protected characteristics, even if such bias is hard to notice for any individual protected characteristic.

### Limitations and further research

The GRADE dataset’s size and richness were extremely useful, but it was not without its disadvantages (particularly within the context of accessing it via the SRS). Given the time and word-limit constraints of this project, it was not feasible to consider every possible combination of protected characteristics of students or to analyse every sub-group. The steps taken during data pre-processing to filter or combine certain categories also reduced the number of sub-groups that could be considered, as well as perhaps the external validity of the results. In particular, the filtering threshold for GCSEs taken (8 + including English and Maths) could be experimented with. The threshold could be important as SES differences in the numbers and types of GCSEs that students take have been previously identified [2]. Future research could take different pre-processing steps and investigate a different subset of the data, as well as take an even more intersectional approach by considering more, and higher order, interactions. The analysis could also be extended to AS- and A-Level students. Moreover, a broader range of models and model configurations could be trialled, with longer training times, to yield a final model with higher predictive accuracy than was obtained in this study.

Indeed, while the large range of pre-processing approaches and models that could be applied to this dataset provide a great deal of flexibility for researchers, they also make it more difficult to interpret results substantively. The CATEs observed in this study are determined not just by the data, but by the models and pre-processing steps themselves. Other models would certainly reveal different CATE values and could result in different findings. The GRADE dataset is certainly rich enough to justify further research and it would be beneficial to either corroborate or repudiate the findings of the current study, by comparing them with the results obtained from a study that employed an intersectional, CATE approach – but with different pre-processing and modelling.

Another potential weakness of this study is the construct validity of some of the variables being used. For example, SES is assessed by proxy through FSM status and IDACI scores here. However, these are more strictly measures of deprivation and cannot really discern relative affluence / high SES – only a lack of deprivation [32]. Similarly, exams necessarily include a measure of a student’s exam-taking ability which may not factor into teacher assessments of academic ability. On the other hand, teachers may assess the attitudinal aspects of their students when creating CAGs [45] that may not be captured by exams. Neither of them can truly measure the latent quality that is a student’s true academic ability. This limits the conclusions of this study as, crucially, bias is not being assessed relative to a perfect baseline but is instead relative to examinations that may already be biased. Additionally, this comparison may also introduce omitted variable bias. Variables such as pupil’s self-motivation, home learning environments and parental aspirations have all been shown to be important for academic attainment [17]. However, given that students spent less time in classrooms/more time at home in 2020, these variables may not have had the same effects in 2018/19 as they did in 2020. Unfortunately, none of these variables are available within the GRADE dataset.

### Conclusion

This paper uses the student- and subject-level GCSE data of the GRADE dataset to formulate the 2020 CAGs as a natural experiment for investigating how teachers’ judgements of academic ability can be biased according to the protected characteristics of their students. A series of models were trained and tested on 2018–19 data from which a tuned LGBM model was selected, due to it having the highest accuracy in its ability to predict grades. This model was then used with the 2020 student data, to produce predictions for what those students were likely to have received had COVID interruptions not occurred, and they had been able to sit their exams. By comparing these modelled results with the teacher assessments that students received, this study estimates the individual treatment effects of the use of teacher assessments on these students. These effects were then summed for each student and averaged across groups and sub-groups of students, delineated by their protected characteristics. This provided average treatment effects for students of those protected characteristics.

Overall, no evidence was found of bias, in absolute terms, against students belonging to any of the protected characteristics considered in this study. In other words, across all groups and sub-groups evaluated here treatment effects were positive—students received higher CAGs than the grades they would have received had they sat their exams as normal. However, there was evidence of relative bias as these treatment effects were not the same for every group and sub-group. Treatment effects were consistently found to be larger for low SES, Chinese and White, no SEN, female and no EAL students. That said, none of these treatment effect differences were substantial when these protected characteristics were investigated individually—particularly if one considers that they must be split up over the 8 + GCSEs that students in this sample took. However, this study also used an intersectional perspective that emphasised the importance of interactions and sub-group differences. The intra-group ranges between groups with the largest and smallest treatment effects were considered at main levels of protected characteristics, two-way interaction levels and three-way interaction levels. The largest intra-group range found at each level increased from main to two-way to a three-way. Indeed, this increase was such that at the three-way level the treatment effects became somewhat substantial – with potentially nearly a half grade point’s difference per subject separating the groups with the largest and smallest treatment effects.

Considering that GCSEs are awarded on a discrete scale, a half-grade point’s difference could have been rounded to a higher or lower CAG because of a student’s protected characteristics. Teacher judgements of academic ability would therefore appear to be somewhat susceptible to intersectional biases, even if biases according to individual protected characteristics are hard to spot. Given what is known about the psychology of bias, this is perhaps unsurprising. Stereotypes are not processed additively by the mind, but instead interact and jointly influence each other in complex and simultaneous ways. An intersectional approach aligns more closely with this theory than approaches that only consider the main effects of protected characteristics. Future quantitative educational equalities research should draw more heavily on the notion of intersectionality. Guidance for teachers on combatting bias should also emphasise the risk of intersectional biases.

## Figures and Tables

**Fig. 13:**
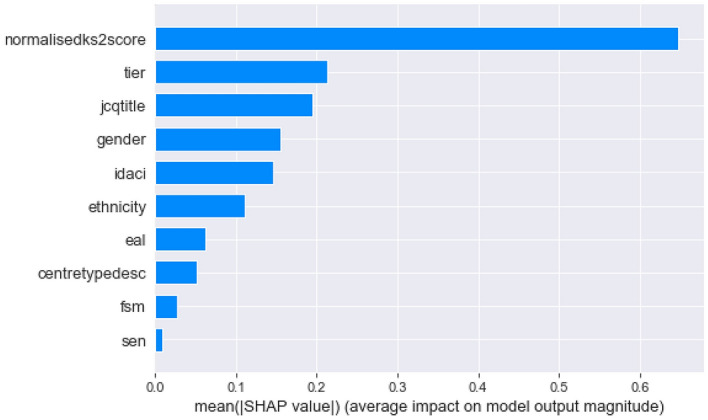
SHAP feature importance bar-plot

**Fig. 14:**
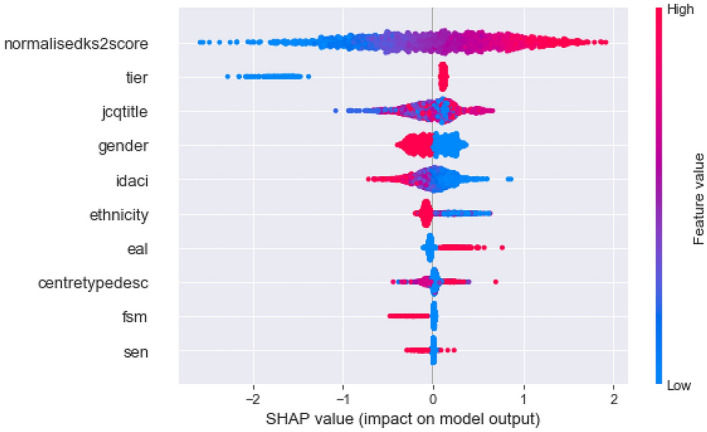
SHAP feature importance summary plot

**Fig. 15:**
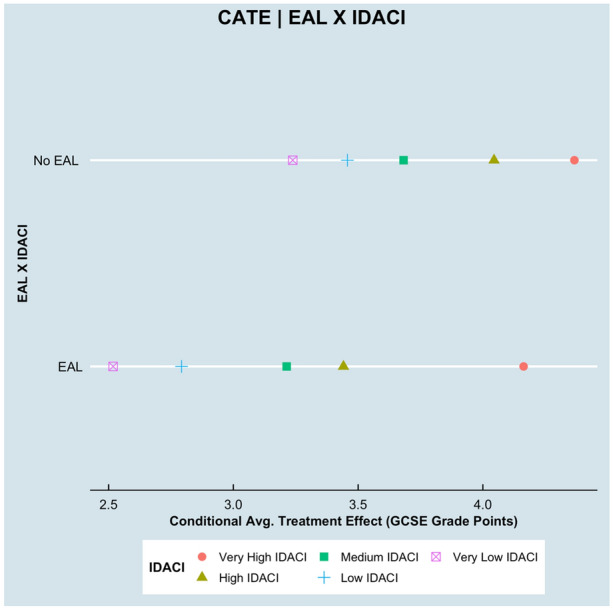
IDACI X EAL

**Fig. 16:**
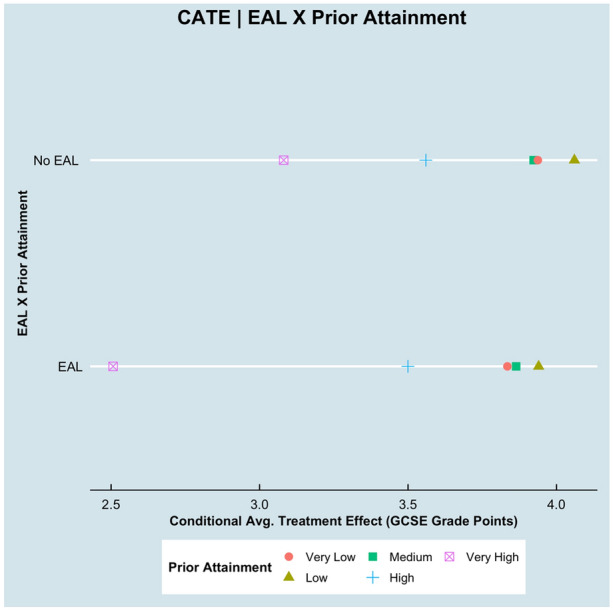
Prior Attainment X EAL

## Data Availability

This work was produced using statistical data from the GRADE dataset accessed via the Office for National Statistics (ONS) Secure Research Service. The use of this data in this work does not imply the endorsement of the ONS in relation to the interpretation or analysis of the statistical data. This work uses research datasets that may not exactly reproduce National Statistics aggregates. The datasets generated and analysed during the current study are not publicly available due to their highly sensitive nature. However, they are available to accredited researchers who make a valid project application: https://www.gov.uk/government/news/data-sharing-framework-for-the-grade-project-published.

## Appendix A

### Subject descriptive statistics

See Table 16.

**Table 16:** GCSE subjects—checking for balance

Variable	Category	Control proportion (%)	Treatment proportion (%)	*p* value
Subject	Art & Design Subjects	2.98	3.20	< 0.001
	Biology	10.28	8.76	< 0.001
	Chemistry	10.28	10.41	< 0.001
	Citizenship Studies	0.28	0.29	< 0.001
	Classical Subjects	0.17	0.24	< 0.001
	Computing	2.33	2.43	< 0.001
	Drama	1.26	1.31	< 0.001
	English Language	10.99	11.22	< 0.001
	English Literature	10.85	11.06	< 0.001
	Food Preparation & Nutrition	0.80	0.80	< 0.001
	French	3.81	3.90	< 0.001
	Geography	5.67	5.70	< 0.001
	German	1.66	1.63	< 0.001
	History	5.87	6.16	< 0.001
	Mathematics	11.04	11.21	< 0.001
	Music	1.08	1.09	< 0.001
	Performing / Expressive Arts	0.20	0.20	< 0.001
	Physical Education	2.04	1.84	< 0.001
	Physics	10.27	10.32	< 0.001
	Religious Studies	5.40	5.19	< 0.001
	Spanish	2.73	3.03	< 0.001

*P* values derived using Chi-squared test. Only GCSEs from either reform phase 1 or 2 considered

## Appendix B

### Feature importance analysis with SHAP

Feature importance analysis using SHAP for the final (LGBM) model was conducted as a sanity check. SHAP assigns each feature an importance value (SHAP value) for a particular prediction [30]. The bar-plot averages the SHAP values across all predictions for each feature, to give an indication of the average impact that feature had on the magnitude of the model’s output. The summary plot takes a random 1000 observations from the treatment data and plots the values they had in each feature against the SHAP values they generated (Figs. 13, 14).

## Appendix C

### Dashboard of full results

The full set of results were uploaded as a dataset into Google BigQuery. A Data Studio dashboard visualising all of them and their intra-group ranges can be found here:

https://datastudio.google.com/reporting/7c49d7ca-ae1c-43cf-a8f7-8e70d969fbad

## Appendix D

### Replication materials

Code used for this project can be found here:

https://github.com/louismagowan/cag-equality

Given the restrictions of the SRS, code had to be put into one notebook and so is less modular and well-organised than it could be. Furthermore, all output within the notebook had to be cleared before it could be released from the SRS, so is blank other than the code itself.

### Accessing the SRS

Due to the highly sensitive nature of this data, access to it is not simple to obtain. Access for this project was only granted after an involved, six-month application process which meant the author had to:

- Study for and pass a “Safe Researcher” examination to become an accredited researcher with the ONS.
- Write three iterations of a project proposal to be reviewed by ethics committees, data owners (DfE, Ofqual) and the ONS research accreditation panel.
- Exclusively work with the data via the ONS SafeRoom in Pimlico, London.
- Submit all research output to a rigorous, two-stage publication clearance process (each level of clearance taking up to five working days) with the ONS Statistical Disclosure team before it could be shared publicly.

Additionally, the SafeRoom through which the SRS was accessed had only limited computational resources, restricting the breadth and number of models that could be trialled (as well as how long they could be tuned for).

## Appendix E

### Further results: IDACI X EAL

Positive, statistically significant CATEs were observed for all categories of IDACI x EAL, as Table 17 demonstrates. The largest CATE was for very high IDACI, no EAL students with 4.367-grade points. The lowest CATE was for very low IDACI, EAL students (2.518). This gave a range of 1.849-grade points. Within both EAL categories, CATEs increased according to IDACI quantile in the exact same order as the IDACI quantiles themselves, i.e., with very low IDACI students having the smallest CATEs and very high IDACI students having the largest CATEs.

As Fig. 15 shows, within each given IDACI quantile, students with no EAL received larger CATEs. These no EAL CATE increases were similar for all IDACI brackets except for very high, which saw a more modest increase moving to no EAL (only 0.204 vs e.g., 0.720 for very low IDACI students).

**Table 17:** CATE | IDACI X EAL

Variable	Category	CATE	Avg. CAG score	Avg. modelled score	*p* value
EAL X IDACI	No EAL X very low IDACI	3.238	61.497	58.259	< 0.001
	No EAL X very high IDACI	4.367	54.859	50.492	< 0.001
	No EAL X medium IDACI	3.682	58.274	54.592	< 0.001
	No EAL X low IDACI	3.457	59.58	56.123	< 0.001
	No EAL X high IDACI	4.045	56.991	52.946	< 0.001
	EAL X very low IDACI	2.518	65.307	62.789	< 0.001
	EAL X very high IDACI	4.163	58.202	54.039	< 0.001
	EAL X medium IDACI	3.214	61.665	58.451	< 0.001
	EAL X low IDACI	2.792	63.602	60.81	< 0.001
	EAL X high IDACI	3.441	59.712	56.271	< 0.001

Same notes as Table 6, except results have been sorted reverse-alphabetically on category

### Further results: prior attainment X EAL

All categories of prior attainment x EAL had positive, statistically significant CATEs (see Table 18). The intra-group range between the largest (no EAL students with low prior attainment, 4.060) and smallest CATEs (EAL students with very high prior attainment, 2.507) was 1.533-grade points.

As seen in Fig. 16, students with no EAL received larger CATEs within each attainment quantile. However, this no EAL increase was much larger for very high-attaining students (an increase of 1.430) than it was for any other attainment quantile (e.g., high only saw an increase of 0.060). Within EAL categories, very high and high-attaining students saw the smallest CATEs. The CATEs of medium, low, and very low quantiles were similar across both EAL categories and did not follow the order of the quantiles themselves.

**Table 18:** CATE | prior attainment X EAL

Variable	Category	CATE	Avg. CAG score	Avg. modelled score	*p* value
Prior attainment X eal	No EAL X very low	3.937	44.832	40.895	< 0.001
	No EAL X very high	3.081	70.08	66.999	< 0.001
	No EAL X medium	3.923	59.198	55.275	< 0.001
	No EAL X low	4.06	54.001	49.941	< 0.001
	No EAL X high	3.56	63.749	60.189	< 0.001
	EAL X very low	3.835	49.326	45.491	< 0.001
	EAL X very high	2.507	72.212	69.705	< 0.001
	EAL X medium	3.864	62.68	58.816	< 0.001
	EAL X low	3.94	57.919	53.979	< 0.001
	EAL X high	3.5	66.862	63.362	< 0.001

Same notes as Table 6, except results have been sorted reverse-alphabetically on category

## Appendix F

### Additional pre-processing steps

Private candidates were removed from the GRADE data since they had not been enrolled at the school or college and had only entered for examinations there. Only 16-year-old^12^ students were considered. Any students who, after joining the results data with the NPD data, still had missing or incomplete observations across any of the variables in Table 2 were also dropped.

### Systematic missingness

Systematic missingness has been previously identified in the GRADE dataset, which would likely have carried through to this study’s sample. For example, in Stratton, Zanini and Noden’s [45] sample over the same years, differences by centre type were identified. Independent schools were found to have the highest proportion of missing data in all categories, accounting for 69% of all missing data at GCSE. Indeed, systematic missingness can probably be found in many of the variables considered. However, since missing data rates are broadly the same across 2018–2020 [27] and this study is making like-for-like comparisons across the years, the impact of missingness should not be too significant. In other words, while between-group differences within a given year might be affected by missingness, changes to those differences over time can be interpreted as changes in outcomes for different groups [27].

## Footnotes

1 Strictly speaking, SES is not a legal protected characteristic—but is included as one for conciseness in this study [16].

2 5 grade points or more.

3 This is in terms of final grades—the higher of either CAGs or Ofqual’s standardised grades (these were equivalent for 94.1% of students) [22].

4 Prior attainment and SES, SES and ethnicity, ethnicity and proportion of non-whites in centre.

5 Such a selection of GCSEs is common within exam results reports, being used in e.g., Attainment 8 / Progress 8 measures [9].

6 Wales and Northern Ireland also administer GCSEs.

7 Due to time complexity of RBF SVR’s being more than quadratic, it had to be trained using a 10% sample of the training data – though it was still used to generate the full set of predictions for the test set.

8 Bearing in mind the statistical disclosure control required by the SRS, sharing results for marginal groups taking less popular GCSEs would not have been possible and observations would have been dropped.

9 Due to concerns about them being disclosive. Certain results were not mentioned in Table 14 and instead were pulled from Appendix C.

10 Other than the main effect for foundation tier, but this is likely an artefact of how foundation tier GCSEs have a cap of 5 discrete grade points and the model is predicting into a continuous space.

11 The largest intra-group range was greater yet again at the four-way interaction level (see Appendix C), though for brevity these results are not discussed.
